# Analysis of Immune and Inflammation Characteristics of Atherosclerosis from Different Sample Sources

**DOI:** 10.1155/2022/5491038

**Published:** 2022-04-25

**Authors:** HAN NIE, Chen Yan, Weimin Zhou, Tao-sheng Li

**Affiliations:** ^1^Department of Stem Cell Biology, Atomic Bomb Diseases Institute, Nagasaki University, China; ^2^Department of Stem Cell Biology, Nagasaki University Graduate School of Biomedical Sciences, 1-12-4 Sakamoto, Nagasaki 852-8523, Japan; ^3^Department of Rheumatology, The Second Affiliated Hospital of Nanchang University, Nanchang City, Jiangxi Province 330006, China; ^4^Department of Vascular Surgery, The Second Affiliated Hospital of Nanchang University, Nanchang City, Jiangxi Province 330006, China

## Abstract

**Background:**

Atherosclerosis is the predominant cause of cardiovascular diseases. Existing studies suggest that the development of atherosclerosis is closely related to inflammation and immunity, but whether there are differences and similarities between atherosclerosis occurring at different sites is still unknown. We elucidated the pathological characteristics of peripheral vascular diseases by using bioinformatic analyses on immune cells and inflammation-related gene expression in atherosclerotic arteries and plaques.

**Methods:**

Eight data sets regarding atherosclerosis were downloaded from the Gene Expression Omnibus database. Human immune genes were obtained from the IMMPORT website. The samples were scored and divided into high- and low-immune groups. Then the samples were analysed using weighted gene co-expression network analysis, while the modules were analysed using functional enrichment. The protein–protein interaction network was constructed using the STRING and Cytoscape databases. The hub immune genes were screened, and the correlation between hub immune genes and immune cells was analysed.

**Results:**

Immune cells and their functions were significantly different during atherosclerosis development. The infiltration proportion of immune cells was approximately similar in samples from different sources of patients with carotid atherosclerosis. However, the sensitivity of lower extremity atherosclerosis samples to immune cells is lower than that of carotid atherosclerosis samples.The samples from the plaque and artery were mainly infiltrated by macrophages, T cells and mast cells. After immune cells were assessed, resting NK cells, activated mast cells and M0 macrophages were found to be key immune cells in atherosclerosis and plaque formation. In addition, CCL4, TLR2, IL1B and PTPRC were considered to be immune marker genes in atherosclerosis development. Conclusion. Bioinformatic data analysis confirms the essential role of immune cells in cardiovascular diseases, and also indicates some differences of immune and inflammation characteristics of atherosclerosis between carotid and lower extremity arteries.

## 1. Introduction

Cardiovascular diseases remain a thorny public health problem in most low- and middle-income countries; however, the morbidity and mortality of cardiovascular diseases have decreased significantly in high-income countries with the improvements in living conditions, medical care and health management [1]. According to the World Health Organization, cardiovascular disease caused 13.2 million deaths in 2011, accounting for 24% of deaths worldwide. Moreover, the death rate due to cardiovascular diseases is expected to reach 23.3 million worldwide by 2030, which promotes cardiovascular diseases as the leading cause of death [2]. Atherosclerosis is a chronic arterial disease that mainly causes most cardiovascular diseases, and its lesions are characterised by the gradual development of lipid deposits in the arterial wall into atheromatous plaques and characteristic plaques. Atherosclerotic plaque formation involves the partial or complete narrowing of the vascular lumen. Moreover, the acute rupture of plaques, a major cause of cerebrovascular disease, leads to local thrombosis and occlusion [3, 4], while plaque formation often leads to peripheral arterial disease. Atherosclerosis used to be considered a lipid metabolism disease [5, 6]; however, increasing experimental and clinical evidence points to atherosclerosis s a chronic immune-inflammatory disease [7–9]. Early-stage atherosclerosis formation involves flow-mediated inflammatory changes in the endothelial cells (ECs), and when ECs are damaged, inflammatory factors such as monocyte chemotactic protein-1, interleukin (IL)-8, vascular adhesion molecule-1, E-selectin and P-selectin are released, which attract lymphocytes and monocytes that infiltrate the arterial wall and lead to inflammation [10]. Following this, macrophages, mast cells, T cells, B cells, tumour necrosis factor (TNF) ILs and other immune cells and inflammatory mediators infiltrate the vessel wall in large numbers to promote atherosclerosis [11, 12]. Currently, the treatment for inflammation has been quite successful, and the strategy for controlling inflammation has been successfully used to treat many other diseases. Therefore, treating inflammation is the preferred target for atherosclerosis treatment. However, the lack of relevant clinical data hinders the effective development of anti-inflammatory drugs for atherosclerosis. Therefore, exploring the immune cells that play an important role in the occurrence and development of atherosclerotic plaque and identifying immune genes that play a key role in inflammation are of great significance for the anti-inflammatory immunotherapy of atherosclerosis.

## 2. Methods

### 2.1. Data Download and Processing

Eight data sets related to atherosclerotic plaque were obtained from the GEO database, namely, GSE21545, GSE24495, GSE28829, GSE163154, GSE43292, GSE24702, GSE100927 and GSE57691 [13–18], which included carotid plaque (N =343), peripheral plaque (N =290), carotid atherosclerotic artery (N = 29) and lower extremity atherosclerotic artery (N = 48). The ‘SVA' package [19] in R software (4.0.2, http://www.r-project.org) was used to remove batch effects and for standardisation of data sets of different platforms.

### 2.2. Single-sample gene set enrichment analysis (ssGSEA) and sample clustering

ssGSEA were performed using GSVA (version 1.34.0) in R software, and the enrichment scores of 29 immune-related cells and their functions in each sample were calculated [20–23]. An unsupervised machine learning method was used to cluster the samples into two clusters. The clusters were further divided into high- and low-immune groups based on their immune scores. Additionally, principal component analysis (PCA) or t-distributed stochastic neighbor embedding (tSNE) was used to reduce the dimension of the grouping data.

### 2.3. Gene Set Enrichment Analysis (GSEA)

The ‘clusterprofiler' package [24] in R software and MSigDB (https://www.gsea-msigdb.org/gsea/msigdb/index.jsp) were used for GSEA analysis in the high- and low-immune groups.

### 2.4. Immune Cell Infiltration and Evaluation

R software was used to identify the cell type by estimating the relative subsets of RNA transcripts (CIBERSORT) [25] and calculate the scores of 22 types of human immune cells in the sample. A *p*-value <0.05 was considered the filtering standard. Differentially expressed immune cells in the high- and low-immune groups were analysed according to the score.

### 2.5. WGCNA

The ‘WGCNA' package [26] in R software was used to analyse the samples based on the Pearson correlation matrix: amn = |cmn *β* (where amn is the proximity between genes m and n, cmn is the Pearson correlation and *β* is the soft power threshold). WGCNA is mainly used for gene co-expression network construction, key disease-related module identification and analysis, protein network construction and key module hub node identification and enrichment analysis.

Gene Ontology (GO) and Kyoto Encyclopedia of Genes and Genomes (KEGG) enrichment analyses.

The enrichment analyses of data obtained from GO and KEGG databases were analysed using the cluster profiler package [24].

### 2.6. Immune-Related Gene Expression Data

The expression profiles of 2498 genes related to immune infiltrating cells, including genes related to antigen-presenting cells, chemokines and their receptors, cytokines and their receptors, interferons and interleukin were downloaded from the ImmPort database (https://www.immport.org/home).

Screening of differentially expressed genes (DEGs) in the high- and low-immune groups.

The ‘limma' package in R software was used to analyse differences in the high- and low-immune groups. The statistical significance of DEGs was set to more than the double change factor (≥1), and the corrected *p*-value error detection rate was <0.05.

### 2.7. Construction of PPI Networks and the Screening of Key Immune Genes

The most immune-related module genes in WGCNA were intersected with the DEGs and immune-related genes. Following this, the STRING database (https://www.string-db.org) was used to construct the PPI network using the intersected genes. The constructed PPI network was inputted into Cytoscape, using the MCC algorithm in cytohubba [27], and the top five genes obtained were identified as key immune genes.

### 2.8. Sensitivity Test and Immune Cell Correlation Test

The ‘pROC' package in R software was used to analyse the subject working characteristic curve of key immune genes, which helped to detect gene sensitivity. The Spearman method was used to detect the correlation between different immune cells and key immune genes.

## 3. Results

### 3.1. Data processing and classification

The procedure of this work shown in the analysis flow chart (Supplementary Figure 1).Based on the sample source and disease type, the samples were divided into plaque samples and artery samples. The plaque samples were further divided into carotid plaque group (343 carotid plaque and 32 control samples; fusion and batch effect of the carotid plaque samples; Supplementary Figure 2A) and peripheral plaque group (290 peripheral plaque samples). The artery samples were further divided into carotid atherosclerosis group (29 carotid atherosclerosis and 12 control samples) and lower limbs atherosclerotic artery group (48 lower limb atherosclerotic artery and 33 control samples; fusion and batch effect of the lower limbs atherosclerotic artery samples; Supplementary Figure 2B). Sample grouping and related information are shown in Table 1.

### 3.2. ssGSEA and Sample Clustering

Atherosclerotic plaque development was speculated to be closely related to immune cells and their function. Therefore, to substantiate this speculation, ssGSEA analysis was performed on GSE28829 (including 16 advanced and 13 early carotid plaque samples), GSE 43292 (including 32 carotid atherosclerotic plaques and 32 control samples) and GSE 100927 (including 29 carotid atherosclerotic artery and 12 control samples). The analysis revealed significant differences in immune cells and their functions between the early and advanced carotid plaque samples, carotid atherosclerotic plaque and control samples, and carotid atherosclerotic artery and control samples (Supplementary Figures 3A, B, C) Further, PCA analysis of the three data sets revealed that the samples in the data sets could be distinguished according to the immune score (Supplementary Figures 3D, E and F), which confirmed the close relation between atherosclerotic plaque development and immune cells and their functions.

**Figure 1:**
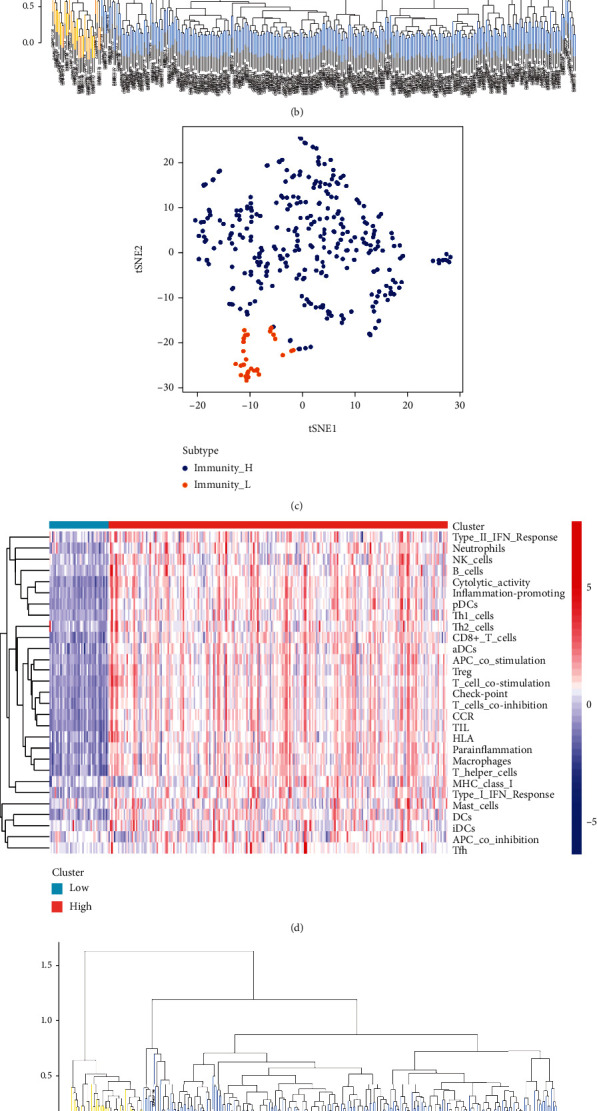
A: Heat map of the carotid artery plaque samples obtained using single-sample gene set enrichment analysis B: Cluster analysis divides the carotid artery plaque samples into the high- and low-immune groups C: t-distributed stochastic neighbor embedding analysis in carotid artery plaque samples. D: Heat map of the peripheral plaque samples obtained using single-sample gene set enrichment analysis E: Cluster analysis divides the carotid artery plaque samples into the high- and low-immune groups F: t-distributed stochastic neighbor embedding analysis in carotid artery plaque samples.

**Figure 2:**
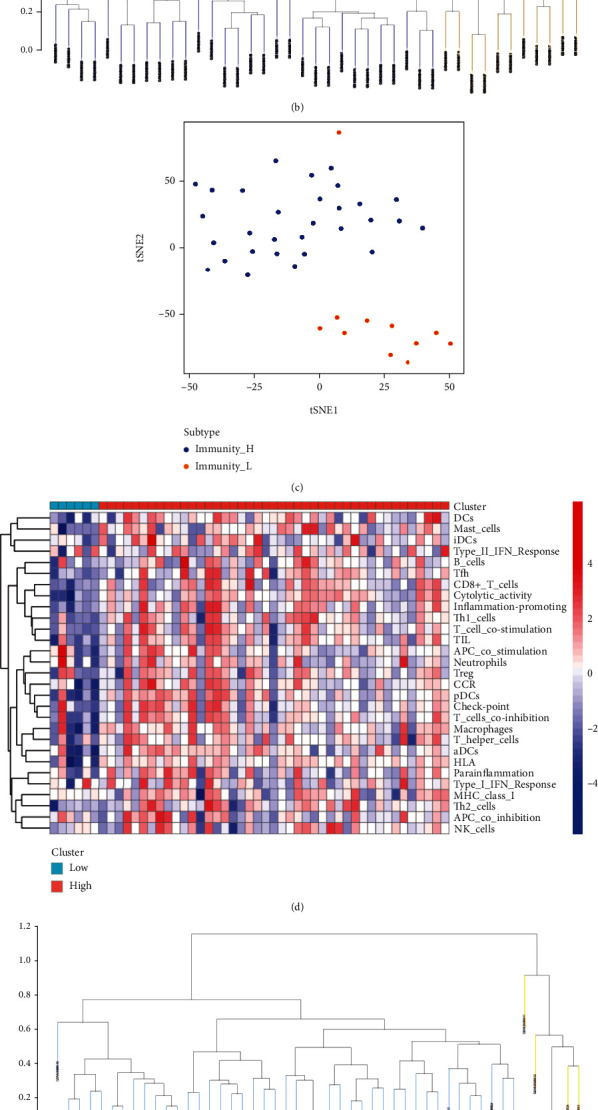
A: Heat map of the carotid atherosclerotic artery samples obtained using single-sample gene set enrichment analysis B: Cluster analysis divides the carotid atherosclerotic artery samples into the high- and low-immune groups C: t-distributed stochastic neighbor embedding analysis in carotid atherosclerotic artery samples D: Heat map of the lower extremity atherosclerotic artery samples obtained using single-sample gene set enrichment analysis E: Cluster analysis divides the lower extremity atherosclerotic artery samples into the high- and low-immunity groups F: t-distributed stochastic neighbour embedding analysis in lower extremity atherosclerotic artery samples.

Therefore, according to the previous grouping, the carotid plaque group was used as an example to obtain ssGSEA scores. The samples were divided into high immune and low immune groups according to the clustering and immune score (Figure 1(a), 1(b)). tSNE analysis was performed according to the immune score and high and low immune group classification, which showed that the samples could be distinguished according to immune score (Figure 1(c)). The other groups, namely, peripheral plaque (Figure 1(d), 1(e), (f)), carotid atherosclerotic artery (Figure 2(a), 2(b), (c)) and lower extremity atherosclerotic artery (Figure 2(d), 2(e), (f)) groups, underwent the same analyses. The tSNE analysis of all groups confirmed that the samples could be divided into high- and low-immune groups according to immune cells and their function.

### 3.3. Differential Gene Analysis and GSEA

**Figure 3:**
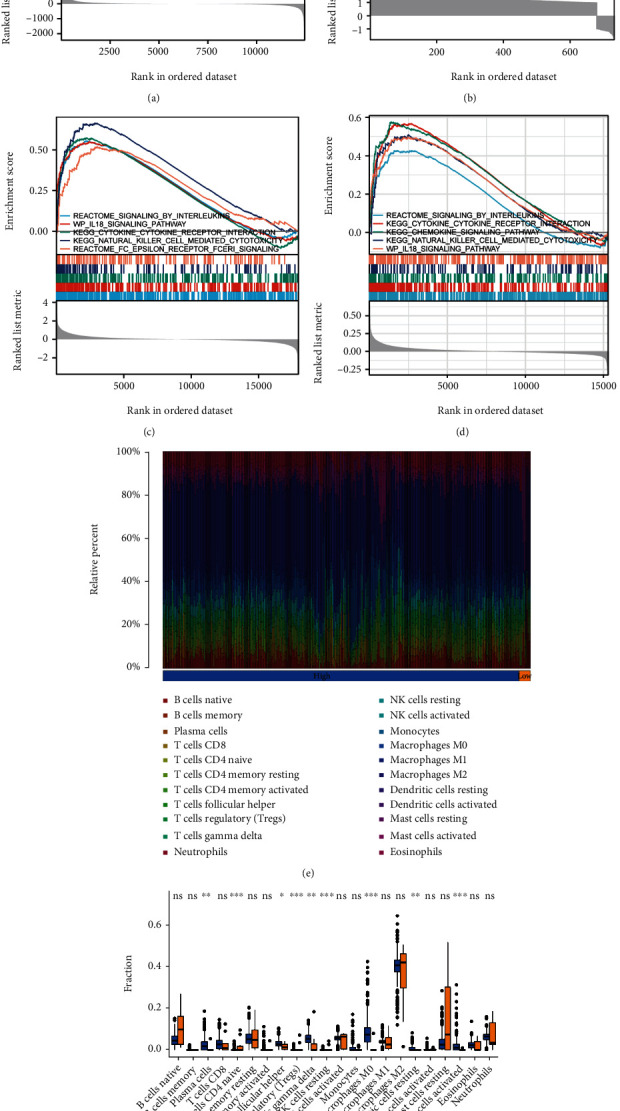
A: Gene set enrichment analysis of the high- and low-immunity groups in carotid artery plaque samples B: Gene set enrichment analysis of the high- and low-immunity groups in peripheral plaque samples C: Gene set enrichment analysis of the high- and low-immunity groups in carotid atherosclerotic artery samples D: Gene set enrichment analysis of the high- and low-immunity groups in lower extremity atherosclerotic artery samples E: The infiltration ratio of 22 immune cells in the carotid artery plaque samples F: The differential expression of 22 immune cells in the high- and low-immune groups in carotid artery plaque samples.

Using the carotid plaque group as an example, 2396 genes were identified as DEGs (LogFC ≥1 or ≤ -1. p < 0.05, Supplementary Figure 4A). GSEA showed that the high- and low-immune groups were closely related to the chemokine signalling pathway and interleukin and inflammatory responses (Figure 3(a)). The same analysis performed on the other groups revealed 772 DEGs in the peripheral plaque group (Supplementary Figure 4B), 1042 DEGs in the carotid atherosclerotic artery group (supplementary Figure 4C) and 68 DEGs in the lower extremity atherosclerotic artery group (Supplementary Figure 4D). Additionally, GSEA showed that the high- and low-immune groups in these types were related to chemokine, interleukin, inflammation pathways and other processes (Figure 3(b); figure 3(c); Figure 3(d)), which further contributes to the credibility of the method to distinguish samples according to immune-related characteristics.

### 3.4. Immune Cell Infiltration and Evaluation

**Figure 4:**
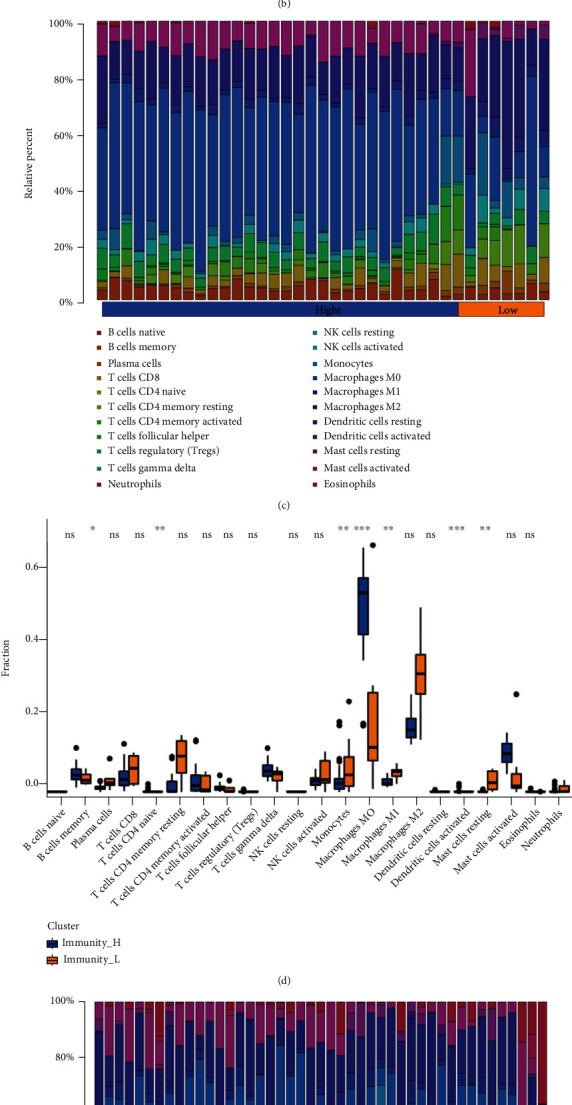
A: The infiltration ratio of 22 immune cells in the peripheral plaque samples B: The differential xpression of 22 immune cells in the high- and low-immune groups in the peripheral plaque sample C: The infiltration ratio of 22 immune cells in the carotid atherosclerotic artery samples D: The differential expression of 22 immune cells in the high- and low-immune groups in the carotid atherosclerotic artery samples E: The infiltration ratio of 22 immune cells in the lower extremity atherosclerotic artery samples F: The differential expression of 22 immune cells in the high- and low-immune groups in the lower extremity atherosclerotic artery samples.

Using the carotid plaque group as an example, CIBERSORT was used to evaluate the infiltration of 22 immune cell types in the group.  Figure 3(e), 3(f) shows that macrophages, mast cells, T cells and B cells mainly infiltrated the carotid plaque. Moreover, significant differences in the expression of nine immune cell types, such as M0 macrophages and activated mast cells, in the high- and low-immune groups, including four T cell types (Figure 3(e), 3(f), Table 2) The same analysis was performed to determine the difference in immune cell infiltration among the peripheral plaque (Figure 4(a), 4(b)), carotid atherosclerotic artery (Figure 4(c), 4(d)) and lower limbs atherosclerotic artery (Figure 4(e), 4(f)) groups. Differences in immune cell expression in the high- and low-immune groups of all samples are shown in Table 2. The results showed that the proportion of immune cells in the peripheral and carotid plaques was roughly the same, with macrophages, especially M2 macrophages, predominately seen in both plaque types (Figure 3(f), Figure 4(f)) Moreover, although the proportion of T cells, NK cells and mast cells was low, they accounted for the majority of immune cell types, such as activated mast cells activated, resting NK cells and CD4 T cells (Table 2).

Macrophages, especially M0 macrophages, were predominantly found in the lower extremity atherosclerotic artery group. Notably, a strong similarity was seen between the differentially expressed immune cells in the lower atherosclerotic artery and plaque groups. The expression of M0 macrophages, M1 macrophages, follicular helper T cells, gamma delta T cells and CD8 T cells was increased in the high-immune group, while the expression of naïve B cells and resting NK cells was increased in the low-immune group (Table 2). Therefore, despite the differences in sample source and sample site, macrophages, T cells and NK cells play a key role in atherosclerosis.

Interestingly, the differentially expressed immune cells of GSE28829 (including 16 advanced and 13 early carotid plaques) and the total carotid plaques group showed great similarity. The expression of M0 macrophages, activated mass cells and gamma delta T cells were increased in the advanced carotid plaque and high-immune groups, while the expression of regulatory T cells (Tregs) was increased in the early carotid plaque and low-immune groups (Supplementary Figure 5A, 5B).

Differentially expressed immune cells in the carotid plaque, peripheral plaque, carotid atherosclerotic artery and lower atherosclerotic artery groups are summarised in Table 2. Immune cells with different expressions in two or more groups, resting NK cells, mast cells, resting CD4 memory T cells, M0 macrophages, CD8 T cells, follicular helper T cells, naïve B cells and plasma cells were screened out. Among them, resting NK cells, M0 macrophages and activated mast cells showed differences in immune cell expression in three groups; therefore, they are considered to play an important role in atherosclerosis development.

### 3.5. WGCNA and Functional Enrichment Analysis

**Figure 5:**
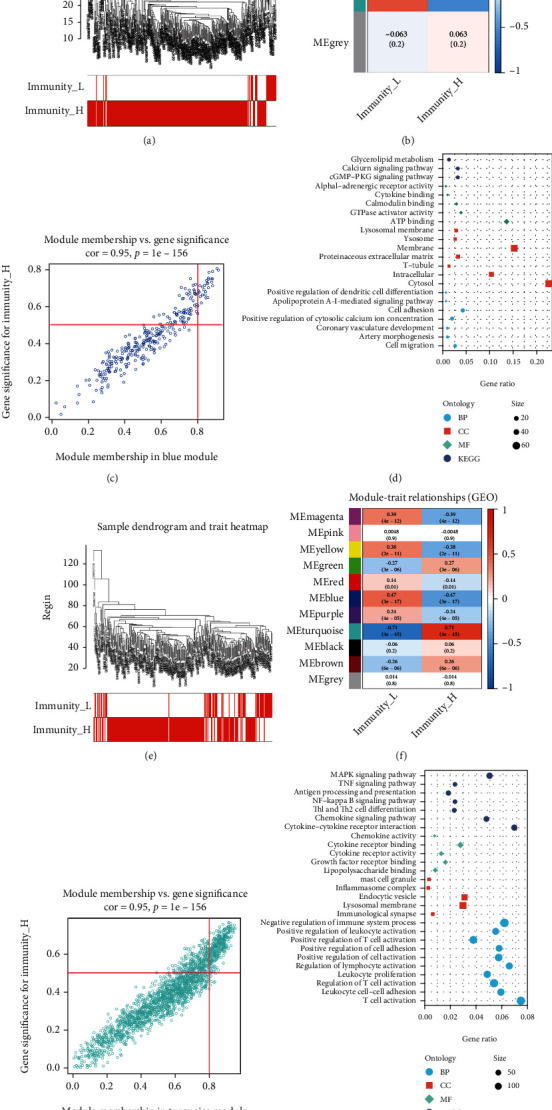
A: Sample clustering dendrogram for detecting outliers in carotid artery plaque samples. B: Correlation diagram between modules and immune groups: each module contains a correlation value (first row) and *p* value (second row) in carotid artery plaque samples. C: The correlation between the genes in the blue module and the high-immunity group in carotid artery plaque samples D: Gene Ontology and Kyoto Encyclopedia of Genes and Genomes enrichment analyses of the genes in the blue module E: Sample clustering dendrogram for detecting outliers in peripheral plaque samples F: Correlation diagram between the modules and immune groups: each module contains a correlation value (first row) and p value (second row) in peripheral plaque samples G: The correlation between the genes in the turquoise module and the high-immunity group in peripheral plaque samples H: Gene Ontology and Kyoto Encyclopedia of Genes and Genomes enrichment analyses of the genes in the turquoise module.

Using the carotid plaque group as an example, samples were analysed using WGCNA according to *β* value to cluster the samples, The *β* value was derived from ‘sft$powerEstimate' choice. (Supplementary Figure 5C). The sample was divided into three modules with *β* = 4 (Figure 5(a), 5(b)), with the blue module showing the highest relation to immunity (Figure 5(c)). The blue module contained 308 genes (Supplementary Table 1), which further underwent GO and KEGG enrichment analyses. The results showed that these genes were related to metabolism, calcium signalling pathway, cell migration and cytokine activation (Figure 5(d)). The same analysis was performed for the other groups.

WGCNA analysis of the peripheral plaque group, with *β* = 6 (Supplementary Figure 5D), divided the group into 11 modules (Figure 5(e), 5(f)). The turquoise module showed the highest relation to immunity (Figure 5(g)) and contained 2120 genes (Supplementary Table 2), which are related to the MAPK pathway, NF-KB pathway, chemokines, cytokines, leukocyte proliferation and T cell activation (Figure 5(h)).

**Figure 6:**
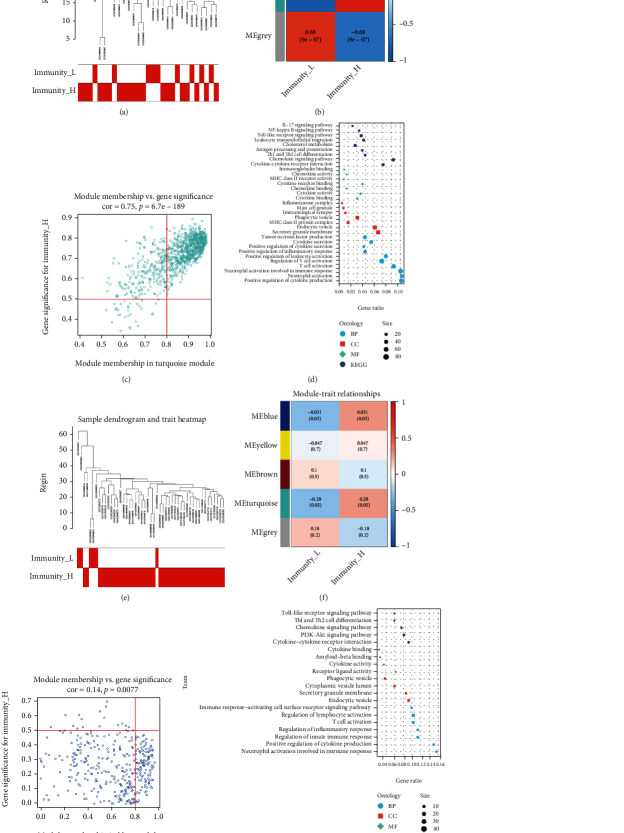
A: Sample clustering dendrogram for detecting outliers in carotid atherosclerotic artery samples B: Correlation diagram between modules and immune groups: each module contains a correlation value (first row) and p value (second row) in carotid atherosclerotic artery samples C: The correlation between the genes in the turquoise module and the high-immunity group in carotid atherosclerotic artery samples D: Gene Ontology and Kyoto Encyclopedia of Genes and Genomes enrichment analyses of the genes in the turquoise module E: Sample clustering dendrogram for detecting outliers in lower extremity atherosclerotic artery samples F: Correlation diagram between the modules and immune groups: each module contains a correlation value (first row) and p value (second row) in lower extremity atherosclerotic artery samples G: The correlation between the genes in the blue module and the high-immunity group in lower extremity atherosclerotic artery samples. H: Gene Ontology and Kyoto Encyclopedia of Genes and Genomes enrichment analyses of the genes in the blue module.

WGCNA analysis of the carotid atherosclerotic artery group, with *β* = 12 (Supplementary Figure 5E), divided the group into three modules (Figure 6(a), 6(b)). The turquoise module showed the highest relation to immunity (Figure 6(c)) and contained 1042 genes (Supplementary Table 3), which are related to THE NF-KB pathway, chemokines, mast cell granules, inflammatory response regulation and neutrophil activation (Figure 6(d)).

WGCNA analysis of the lower extremity atherosclerotic artery group, with *β* = 5 (Supplementary Figure 5F), divided the group into five modules (Figure 6(e), 6(f)). The blue module showed the highest relation to immunity (Figure 6(g)) and contained 361 genes (Supplementary Table 4), which are related to the toll-like receptor signalling pathway, Akt signalling pathway, chemokine signalling pathway, cytokine activity and T cell activation pathway (Figure 6(h)).

### 3.6. Construction of a PPI Network and Screening of Key Immune Genes

**Figure 7:**
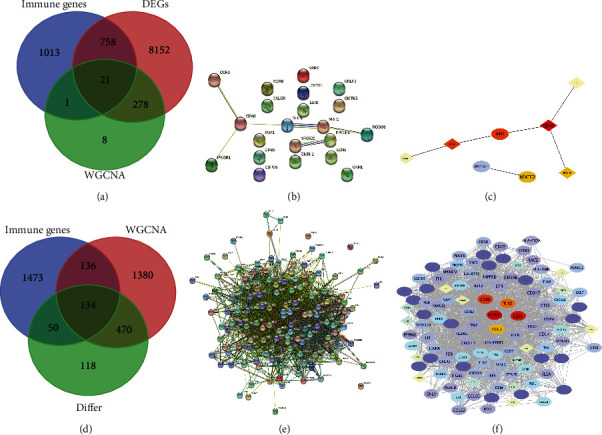
A: Venn diagram of immune-related genes, differentially expressed genes and blue modular genes in carotid artery plaque samples B: Protein-protein interaction network construction using 21 genes from the STRING database in carotid artery plaque samples C: Screening of the top five hub genes (the darker the colour, the higher the gene ranking) in carotid artery plaque samples D: Venn diagram of immune-related genes, differentially expressed genes and turquoise modular genes in peripheral plaque samples E: Protein–protein interaction network construction using 134 genes from the STRING database in peripheral plaque samples F: Screening of the top five hub genes (the darker the colour, the higher the gene ranking) in peripheral plaque samples.

**Figure 8:**
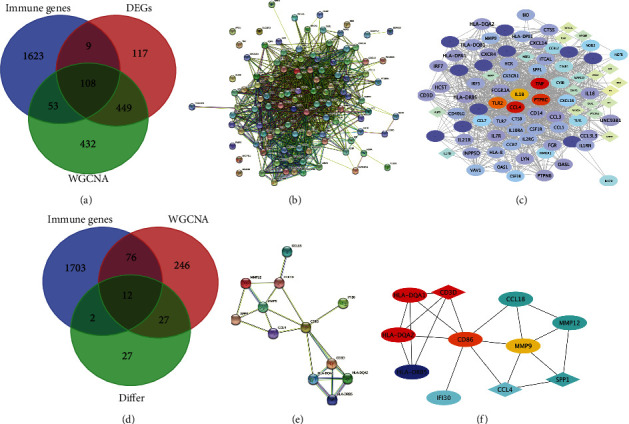
A: Venn diagram of immune-related genes, differentially expressed genes and turquoise modular genes in the carotid atherosclerotic artery samples B: Protein–protein interaction network construction using 108 genes from the STRING database in the carotid atherosclerotic artery samples C: Screening of the top five hub genes (the darker the colour, the higher the gene ranking) in the carotid atherosclerotic artery samples D: Venn diagram of immune-related genes, differentially expressed genes and blue module genes in lower extremity atherosclerotic artery samples E: Protein–protein interaction network construction using 12 genes from the STRING database in lower extremity atherosclerotic artery samples F: Screening of the top five hub genes (the darker the colour, the closer the gene ranking) in lower extremity atherosclerotic artery samples.

Using the carotid plaque group as an example, the blue module genes, which were closely related to immunity in WGCNA, were intersected with the high and low immune group genes and immune-related genes to obtain 21 intersecting genes (Figure 7(a)). These genes were introduced into the STRING website for PPI network construction (Figure 7(b)). Following this, the constructed PPI network was introduced into Cytoscape and analysed using the MCC algorithm in cytohubba to screen for the top five genes: CD40, NRP1, NRP2, NFATC2, IFNGR1 (Figure 7(c)). The same analysis was performed for the other groups. A total of 134 intersecting genes were obtained in the peripheral plaque group (Figure 7(d)), with PTPRC, CD4, CCL2, TLR2 and CD86 identified as key immune genes (Figure 7(e), 7(f)). A total of 108 intersecting genes were obtained in the carotid atherosclerotic artery group (Figure 8(a)), with TNF, PTPRC, CCl4, TLR2 and IL1B identified as key immune genes (Figure 8(b), 8(c)). A total of 12 intersecting genes were obtained in the lower extremity atherosclerotic artery group (Figure 8(d)), with HLA-DQA1, HLA-DQA2, CD3D, CD86 and MMP9 identified as key immune genes (Figure 8(e), 8(f)).

### 3.7. Correlation Analysis of Immune Cells

**Figure 9:**
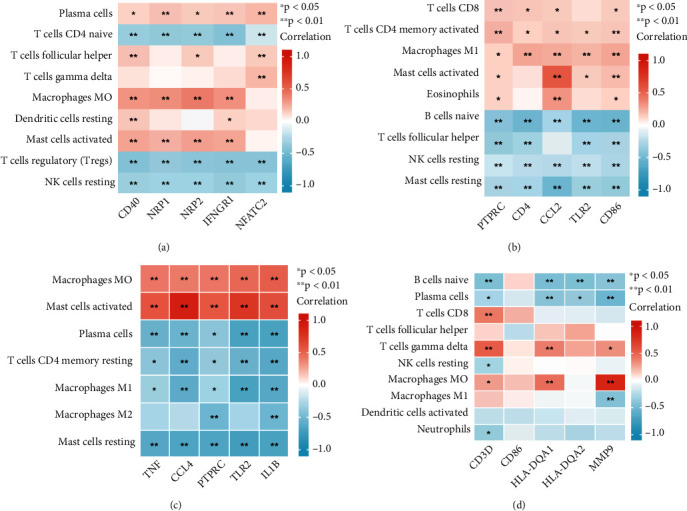
A: Heat map of the correlation between five hub genes and differentially expressed immune cells in carotid artery plaque samples B: Heat map of the correlation between five hub genes and differentially expressed immune cells peripheral plaque samples C: Heat map of the correlation between five hub genes and differentially expressed immune cells in the carotid atherosclerotic artery samples D: Heat map of the correlation between five hub genes and differentially expressed immune cells in lower extremity atherosclerotic artery samples.

The correlation between the key immune genes screened in each group and differentially expressed immune cells of the corresponding groups were analysed: carotid plaque (Figure 9(a)), peripheral plaque (Figure 9(b)), carotid atherosclerotic artery (Figure 9(c)) and lower extremity atherosclerotic artery (Figure 9(d)) groups. The results are shown in Supplementary Table 5. Although most immune genes were correlated with immune cells (p < 0.05), the correlation was not very high (r < 0.5). Moreover, compared with the plaque and peripheral blood groups, the correlation between immune genes and immune cells in the artery group was very high (r > 0.5), especially the correlation between *CCL4* and activated mast cells was as high as 0.9 (Figure 9(c)). r > 0.5 was used as the standard to screen the key immune genes closely related to immune cells, which were then analysed according to the situation of each group. Since *PTPRC* and *TLR2* were common key immune genes in the PPI network of groups with strong correlations with most immune cells in both groups, they were identified as hub immune genes that were closely related to immune cells. Based on the correlation criteria and correlation criteria value, *CCL2*, *CCL4, TLR2, IL1B* and *PTPRC* were identified as hub immune genes in atherosclerosis.

### 3.8. Receiver Operating Characteristic Curve Analysis

**Figure 10:**
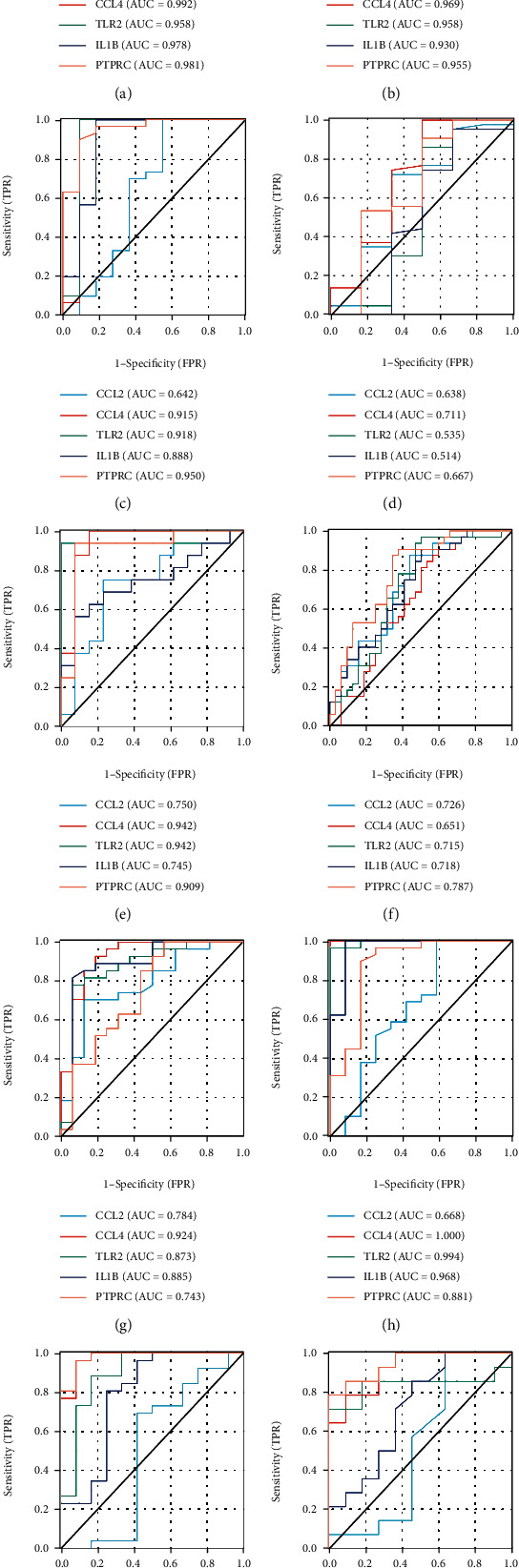
A: ROC analysis of CCL2, CCL4, TLR2, IL1B and PTPRC in the carotid plaque group B: ROC analysis of CCL2, CCL4, TLR2, IL1B and PTPRC in the peripheral plaque group C: ROC analysis of CCL2, CCL4, TLR2, IL1B and PTPRC in the lower extremity atherosclerotic artery group D: ROC analysis of CCL2, CCL4, TLR2, IL1B and PTPRC in the artery group E: ROC analysis of CCL2, CCL4, TLR2, IL1B and PTPRC in GSE28829 (including 16 advanced and 13 early carotid plaques) F: ROC analysis of CCL2, CCL4, TLR2, IL1B and PTPRC in GSE43292 (including 32 carotid plaques and 32 control samples) G: ROC analysis of CCL2, CCL4, TLR2, IL1B and PTPRC in GSE16315 (including 27 intraplaque and 16 non intraplaque haemorrhage samples) H: ROC analysis of CCL2, CCL4, TLR2, IL1B and PTPRC in GSE100927 (including 29 carotid atherosclerotic artery samples and 12 control samples) I: ROC analysis of CCL2, CCL4, TLR2, IL1B and PTPRC in GSE100927 (including 25 femoral atherosclerotic artery samples and 12 control samples) J: ROC analysis of CCL2, CCL4, TLR2, IL1B and PTPRC in GSE100927 (including 14 infra-popliteal atherosclerotic artery samples and 11control samples).

To verify the reliability of the selected key immune genes, the receiver operating characteristic curve(ROC) analysis of CCL2, CCL4, TLR2, IL1B and PTPRC in the plaque and artery groups were analysed. The results showed that the five genes had high accuracy in all groups except the lower extremity atherosclerotic artery group (Figure 10(a), 10(b), (c), (d)). To further verify the accuracy of the genes, ROC analysis was performed on these five genes in the GSE28829 (including 16 advanced and 13 early carotid plaques), GSE163154 (including 27 intraplaque and 16 non intraplaque haemorrhage samples), GSE43292 (including 32 carotid plaques and 32 control samples), GSE100927 (including 29 carotid atherosclerotic artery samples and 12 control samples), GSE100927 (including 25 femoral atherosclerotic artery samples and 12 control samples) and GSE100927 (including 14 infra-popliteal atherosclerotic artery samples and 11 control samples) datasets. These five genes showed strong accuracy in identifying carotid plaque progression (Figure 10(e)), plaque stability (Figure 10(f)) and carotid atherosclerosis and plaque occurrence (Figure 10(g), (h)), which indicates the close relationship of these five immune genes with atherosclerosis and plaque occurrence and plaque progression and stability. However, the accuracy of these five genes in the lower extremity atherosclerotic artery group was lower than that of the other three groups. Therefore, the lower extremity atherosclerotic artery group was further subdivided into the following groups: femoral artery and inferior popliteal artery. The verification of these subgroups showed the strong accuracy of the other four genes except CCL2 (Figure 10(i), (j)). However, differences were observed in immune responses between the lower extremity atherosclerosis, carotid atherosclerosis and atherosclerotic plaque groups. Moreover, the distinguishing accuracy between the high- and low-immune groups is lower than that of the other sample groups. In addition to CCL2, the four genes (CCL4, TLR2, IL1B and PTPRC) can distinguish between lower extremities atherosclerotic artery and lower extremities normal artery. Although CCL2 does not perform well in the lower extremity atherosclerotic artery, it performs well in the carotid atherosclerotic artery and plaque groups, which indicates that CCL2 is a specific immune gene for carotid atherosclerosis and plaque formation.

## 4. Discussion

To the best of our knowledge, this study is the first to comprehensively analyse and compare the immune characteristics of four different atherosclerosis sample types: carotid plaque, peripheral plaque, carotid atherosclerotic artery and lower extremity atherosclerotic artery.

Comprehensive analyses showed that despite differences in sample source, the immune cell infiltration in atherosclerotic plaque and artery samples were dominated by macrophages, especially in the carotid plaque and carotid atherosclerotic artery groups. M0 macrophages accounted for a large proportion of the infiltered cells and their expression in the high immune group was significantly higher than that in the low immune group. Macrophages play a central role in atherosclerosis as inflammation regulators. The main cells involved in atherosclerotic plaque formation are activated macrophages and foam cells, with macrophages promoting two key changes in plaque morphology: plaque necrosis and protective collagen scar (fibrous cap) thinning [28]. A study by Ginhoux et al. [29] showed that macrophages in the plaque can induce high inflammation tissue destruction. M0 macrophages are usually considered to be inactivated macrophages and are induced by IFN-*γ*, TLR4, IL-4, IL-13 and other factors, thus differentiating into pro-inflammatory M1 macrophage or anti-inflammatory M2 macrophage [30, 31]. Therefore, inducing macrophage differentiation may control atheroma, which is a key factor in the development of hardening.

Despite being present in smaller proportion as compared to macrophages, T cells, NK cells and mast cells showed strong heterogeneity in the high- and low-immunity group, especially T cells. T cell expression varied between the high- and low-immunity groups in plaque, artery and lower extremity atherosclerotic artery source samples. Among the differentially expressed T cells, CD4 memory T cells and CD8 T cells were dominant. Previous studies have shown that most T cells found in atherosclerotic plaques show memory phenotype and have large CD4 T cell numbers. After stimulation by antigens, stimulants and some cytokines, CD4 T cells differentiate into different T cell subsets and participate in the immune and inflammatory processes [32, 33]. Although CD8 T cells are rare compared to CD4 T cells in atherosclerotic plaques, CD8 T cells showed a significant increase in severe atherosclerotic plaque lesions, indicating that CD8 T cells are inextricably linked with plaque progression and inflammation. Additionally, some studies have reported that CD8 T cells contribute to plaque inflammation and necrotic core formation, suggesting their role in promoting plaque instability and causing cardiovascular events after plaque rupture [34–37]. Moreover, these studies have indicated that T cells, especially CD4 memory T cells and CD8 T cells, can be the key immune cells that promote atherosclerosis formation and plaque progression, which is consistent with this study. Notably, Tregs only showed high expression in the low-immune group in carotid plaque samples in comparison with other differential immune cells. Tregs also showed a higher correlation with key genes than the other immune cells, and Tregs expression increased in the early carotid plaque group compared with the advanced carotid plaque group. Liu et al. reported that the number of Tregs in the unstable plaques of human origin was reduced. Correspondingly, studies in animal models have also demonstrated that Tregs can secrete anti-inflammatory cytokine IL-10 and TGF in stable plaque- *β* and inhibit pro-inflammatory T cell proliferation, delaying atherosclerosis occurrence and development [38–40]. Further evidence suggests that Tregs promote the transformation from pro-inflammatory M1 macrophages to anti-inflammatory M2 macrophages by releasing IL-10 to prevent atherosclerosis occurrence. Therefore, Tregs play an anti-inflammatory role in carotid plaque and delay plaque progression.

The role of mast cells should not be underestimated despite their lower content in atherosclerosis and plaque compared to macrophages. Mast cells are mainly present in the mucosal and connective tissues and distributed around the blood vessels, activating the release of proinflammatory cytokines, vasoactive substances and proteolytic enzymes. These characteristics of mast cells have extremely strong effects on the blood vessels [41, 42]. This study showed that activated mast cell numbers increased in the high-immune groups of the plaque and artery samples, showing a strong correlation between the screened key genes and activated mast cells. Wang et al. confirmed that mast cell density and degranulated cell proportion in the shoulder area of the carotid plaque were significantly higher than those in other areas, and mast cells were also found around plaque thrombosis, calcification and neovascularization, indicating their role in carotid atherosclerotic plaque formation and affecting plaque stability by activating degranulation [43]. Various studies have shown that mast cells located near plaque microvessels contain basic fibroblast growth factor (bFGF). Mast cells release angiogenesis-related compounds via bFGF, which enhances the ability of the matrix around cells to degrade protease, induces the leakage and rupture of neovascularization and leads to bleeding in plaque [44]. Den et al. reported that activated mast cells release chymotrypsin via the TLR4 pathway and promote vascular smooth muscle cell apoptosis in the body, which leads to plaque instability [45]. Therefore, previous reports and the current study mutually confirm that inhibiting mast cell activation is a potential therapeutic target and an important research direction for controlling atherosclerosis development and plaque stabilisation. However, the role of NK cells in atherosclerosis remains unclear. Selathurai et al. [46] speculated that perforin and granzyme B produced by NK cells can promote atherosclerosis and necrotic core development, whereas Nour-Eldine et al. [47] speculated that NK cells have no direct effect on hypercholesterolaemia induced atherosclerosis. However, NK cell may promote atherosclerosis formation when pathological NK cells are over-activated. This study shows that in the low-immunity group of the carotid and peripheral plaques, resting NK cell expression increased significantly and resting NK cells number in the early carotid plaque increased compared with the late carotid plaque. Therefore, NK cells contribute to atherosclerosis formation. Interestingly, we found that B-naïve cells were highly expressed in the low immune group of the peripheral plaques and lower limbs atherosclerotic arteries, but not in the carotid plaque and carotid atherosclerotic artery. In contrast to T cells and macrophages, the role of B cells in atherosclerosis has only recently begun to receive attention. Recent studies have shown that B cell production is significantly reduced in the bone marrow of aged mice and humans [48], Frasca et al. found that aging downregulates B cell type switching in humans and mice [49]; in addition, hypercholesterolemia Apoe-/- mice and low-density lipoprotein-deficient mice have also shown that protective immunity of B cells can delay the progression of atherosclerosis, while the lack of B cells increases the occurrence of atherosclerosis [50, 51]. Further studies have found that the lack of B cell in mice can lead to vascular endothelium dysfunction by inducing the increase of neutrophils, thereby leading to vascular diseases [52]. These studies demonstrate the importance of B cells in the development of atherosclerosis and may have a more significant impact on large vessel atherosclerosis than in carotid atherosclerosis, but more work is needed to confirm this speculation.

Bioinformatic and correlation analyses of differentially expressed immune cells helped to identify CCL4, TLR2, IL1B, and PTPRC as hub genes in atherosclerosis formation and plaque progression. CCL2 affects carotid atherosclerosis and plaque formation. CCL2 and CCL4 are both pro-inflammatory chemokines in the chemokine family, which attract immune cells to the inflammation site and actively participate in the inflammatory response under the stimulation of IL-1, TNF-*α* and LPS. Previous studies have shown that CCL2 inhibition alone or in combination with CX3CR1 and CCR5 leads to decreased monocyte recruitment and thus inhibits atherosclerosis occurrence. Winter et al. reported that blocking the CCL2-CCR2 axis can effectively reduce atherosclerosis occurrence. [53, 54]. However, inhibiting CCL4 can reduce MMP-2 and MMP-9 expression, which blocks TNF-*α* and IL-6 production that is responsible for stimulating macrophage activation and inhibits macrophage and EC activation to reduce atherosclerotic plaque formation and increase plaque stability [55]. Therefore, CCL2 and CCL4 are novel therapeutic targets for regulating atherosclerosis. Notably, CCL4 has the highest correlation with mast cells in this study. Therefore, exploring the interaction between CCL4 and mast cells may provide new insights into atherosclerosis treatment. As a member of innate immune toll-like receptor (TLR), TLR2 has been proved to play an important role in the innate immune mechanism. Recent studies have indicated that TLR2 is actively involved in atherosclerosis caused by Chlamydia pneumoniae, Porphyromonas gingivalis and Helicobacter pylori [56–58]. Studies have shown that certain drugs blocked TLR2 in ApoE(-/-) mice, which causes the expression of pro-inflammatory cytokines IL-6 and TNF-*α* to decrease and inactivate NF-KB and STAT3, and thereby greatly reducing plaque area and vascular stenosis [59]. Furthermore, TLR2 can induce chondrogenesis in vascular smooth muscle cells through osteoprotegerin and IL-6-mediated RANKL, leading to vascular calcification and thus promoting atherosclerosis formation [60]. Everett et al. reported a gratifying result by using IL1B inhibitor canakinumab to effectively inhibit inflammation and improve cardiovascular disease outcomes, suggesting that inflammation is a key factor driving atherosclerosis and targeting immune inflammation. Targeted therapy of related genes has broad prospects regarding atherosclerosis treatment [61]. PTPRC (also known as CD45) is an important leukocyte antigen involved in the immune regulation of T and B cells. It has been identified as a hub gene in several studies on cardiovascular diseases [62–64].

In this study, we found that compared with carotid atherosclerotic plaque samples, the correlation between lower extremity atherosclerosis samples and immune cells was low, and the sensitivity of key genes to lower extremity atherosclerosis immune groups was also reduced. According to previous studies, carotid atherosclerotic plaques show a high incidence and early onset compared with lower extremity arteries [65, 66]. This also indirectly supports the occurrence of carotid atherosclerotic plaque, which may be more closely related to immune inflammation. Therefore, the treatment related to immune inflammation may be more effective for carotid atherosclerosis.

Although previous studies have analysed key genes of atherosclerosis-related diseases [67–70], large sample bioinformatic analyses for different atherosclerosis tissue sources and plaque formation are yet to be reported. This study identified the immune cell infiltration and hub gene in atherosclerosis samples from different tissue sources (plaque, artery) and different sites (carotid plaque, peripheral plaque and lower extremity), which helps to further explain immune cell effects and inflammatory processes on atherosclerosis and the similarities and differences between atherosclerosis samples from different sources. However, this study has some limitations. First, although more than 700 samples were analysed, this study focussed on carotid, peripheral and lower extremity atherosclerotic diseases but did not include coronary atherosclerosis, myocardial infarction and other diseases. Second, this is a retrospective study; therefore, a prospective study design or larger sample size can help to further verify the function of hub genes.

## 5. Conclusion

This study not only presents new ideas regarding atherosclerosis formation and plaque progression but also provides potential therapeutic targets for atherosclerotic arteries and plaques treatment.

## Figures and Tables

**Table 1:** Dataset information.

Plaque(series)	Platform	Samples	Group
21545	570	Carotid plaque 126	Carotid plaque
24495	10687	Carotid plaque 113	
28829	570	Carotid plaque 29 (advanced 16; early 13)	
163154	6104	Carotid plaque 43 (non-IPH 16; IPH 27)	
43292	6244	Carotid plaque 32; control samples 32	
24702	10687	Peripheral plaque 290	Peripheral plaque
Artery	Platform	Samples	Group
100927	17077	Carotid atherosclerotic artery:29; carotid artery control:12	Carotid atherosclerotic artery
100927	17077	Femoral atherosclerotic artery:25; femoral artery control: 12	Lower extremity atherosclerotic artery
100927	17077	Infra-popliteal atherosclerotic artery:14; infra-popliteal artery control: 11	
57691	10558	Lower extremity aortic occlusive disease:9; control artery:10	

**Table 2:** Differentially expressed immune cells in each group (the immune cells with increased expression in each group are listed in the table).

Group	Immunity-High	Immunity-Low
Carotid plaque	Plasma cells	T cells regulatory (Tregs)
	T cells CD4 naive	NK cells resting
	T cells follicular helper	
	T cells gamma delta	
	Macrophages M0	
	Dendritic cells resting	
	Mast cells activated	
Peripheral plaque	T cells CD8	B cells naive
	T cells CD4 memory activated	T cells follicular helper
	Macrophages M1	NK cells resting
	Mast cells activated	Mast cells resting
	Eosinophils	
Carotid atherosclerotic artery	Macrophages M0	Plasma cells
	Mast cells activated	T cells CD4 memory resting
		Macrophages M1
		Macrophages M2
		Mast cells resting
Lower limbs atherosclerotic arteries	T cells CD8	B cells naive
	T cells follicular helper	Plasma cells
	T cells gamma delta	NK cells resting
	Macrophages M0	Dendritic cells activated
	Macrophages M1	Neutrophils

## Data Availability

The datasets used and/or analyzed during the current study are available from the corresponding author on reasonable request.

## Supplementary Materials

Supplementary Materials Supplementary Figure1: Analysis flow chart of this work. Supplementary Figure 2. A: The fusion and de-batch effect of five carotid artery plaque data sets B: The fusion and de-batch effect of two lower extremity atherosclerotic artery data sets. Supplementary Figure 3 A: Heatmap of GSE28829 (including 16 advanced and 13 early carotid plaques) obtained using single-sample gene set enrichment analysis (ssGSEA) B: Heatmap of GSE43292 (including 32 carotid plaques and 32 control samples) obtained using ssGSEA C: Heatmap of GSE100927 (including 29 carotid atherosclerotic artery samples and 12 control samples) obtained using ssGSEA D: Principal component analysis (PCA) of GSE28829 (according to ssGSEA score) E: PCA analysis of GSE43292 (according to ssGSEA score) F: PCA analysis of GSE100927 (according to ssGSEA score). Supplementary Figure 4 A: The volcano map of the differences in gene analysis between the high- and low-immune groups in carotid plaque samples B: The volcano map of the differences in gene analysis between the high- and low-immune groups in peripheral plaque samples C: The volcano map of the differences in gene analysis between the high- and low-immune groups in carotid atherosclerotic artery samples D: The volcano map of the differences in gene analysis between the high- and low-immune groups in lower extremity atherosclerotic artery samples. Supplementary Figure 5 A: Proportion of 22 types of immune cell infiltration in GSE28829 (including 16 advanced and 13 early carotid plaques) B: Differential expression of 22 immune cells in GSE28829 (including 16 advanced and 13 early carotid plaques) between the high and low immune groups C: Selection process of the soft threshold using weighted gene co-expression network analysis (WGCNA) in the carotid plaque group D: Selection process of the soft threshold using WGCNA in the peripheral plaque group E: Selection process of the soft threshold using WGCNA in the carotid atherosclerotic artery group F: Selection process of the soft threshold using WGCNA in the lower extremity atherosclerotic artery group. Supplementary Materials Supplementary Table 1: three hundred and eight genes in the blue module in carotid plaque group Supplementary Table 2: two thousand one hundred and twenty genes in the turquoise module in the peripheral plaque group Supplementary Table 3: one thousand and forty-two genes in the turquoise module in the carotid atherosclerotic artery group Supplementary Table 4: three hundred and sixty-one genes in the blue module in the lower extremity atherosclerotic artery group Supplementary Table 5:Correlation analysis between differential immune cells and hub gene in all groups (r value; p value).

## References

[1] Herrington W., Lacey B., Sherliker P., Armitage J., Lewington S. (2016). Epidemiology of atherosclerosis and the potential to reduce the global burden of Atherothrombotic disease. *Circulation Research*.

[2] World Health Organization (2013). *Media centre, Cardiovascular diseases (CVDs)*.

[3] Libby P. (2002). Inflammation in atherosclerosis. *Nature*.

[4] Kanter J. E., Kramer F., Barnhart S. (2012). Diabetes promotes an inflammatory macrophage phenotype and atherosclerosis through acyl-CoA synthetase 1. *Proceedings of the National Academy of Sciences*.

[5] Tabas I., Williams K. J., Borén J. (2007). Subendothelial lipoprotein retention as the initiating process in atherosclerosis: update and therapeutic implications. *Circulation*.

[6] Camejo G., Lalaguna F., López F., Starosta R. (1980). Characterization and properties of a lipoprotein-complexing proteoglycan from human aorta. *Atherosclerosis*.

[7] Ross R. (1999). Atherosclerosis--an inflammatory disease. *The New England Journal of Medicine*.

[8] Libby P., Ridker P. M., Hansson G. K. (2011). Progress and challenges in translating the biology of atherosclerosis. *Nature*.

[9] Tabas I., García-Cardeña G., Owens G. K. (2015). Recent insights into the cellular biology of atherosclerosis. *The Journal of Cell Biology*.

[10] Chistiakov D. A., Melnichenko A. A., Grechko A. V., Myasoedova V. A., Orekhov A. N. (2018). Potential of anti-inflammatory agents for treatment of atherosclerosis. *Experimental and Molecular Pathology*.

[11] Libby P. (2001). Current concepts of the pathogenesis of the acute coronary syndromes. *Circulation*.

[12] Kaartinen M., Penttilä A., Kovanen P. T. (1994). Mast cells of two types differing in neutral protease composition in the human aortic intima. Demonstration of tryptase- and tryptase/chymase-containing mast cells in normal intimas, fatty streaks, and the shoulder region of atheromas. *Arteriosclerosis and Thrombosis*.

[13] Folkersen L., Persson J., Ekstrand J. (2012). Prediction of ischemic events on the basis of transcriptomic and genomic profiling in patients undergoing carotid endarterectomy. *Molecular Medicine*.

[14] Puig O., Yuan J., Stepaniants S. (2011). A gene expression signature that classifies human atherosclerotic plaque by relative inflammation status. *Circulation. Cardiovascular Genetics*.

[15] Döring Y., Manthey H. D., Drechsler M. (2012). Auto-antigenic protein-DNA complexes stimulate plasmacytoid dendritic cells to promote atherosclerosis. *Circulation*.

[16] Jin H., Goossens P., Juhasz P. (2021). Integrative multiomics analysis of human atherosclerosis reveals a serum response factor-driven network associated with intraplaque hemorrhage. *Clinical and Translational Medicine*.

[17] Ayari H., Bricca G. (2013). Identification of two genes potentially associated in iron-heme homeostasis in human carotid plaque using microarray analysis. *Journal of Biosciences*.

[18] Steenman M., Espitia O., Maurel B. (2018). Identification of genomic differences among peripheral arterial beds in atherosclerotic and healthy arteries. *Scientific Reports*.

[19] Leek J. T., Evan Johnson W., Parker H. S. (2020). Sva: surrogate variable analysis. *R package version*.

[20] Hänzelmann S., Castelo R., Guinney J. (2013). GSVA: gene set variation analysis for microarray and RNA-seq data. *BMC Bioinformatics*.

[21] Yue C., Ma H., Zhou Y. (2019). Identification of prognostic gene signature associated with microenvironment of lung adenocarcinoma. *PeerJ*.

[22] He Y., Jiang Z., Chen C., Wang X. (2018). Classification of triple-negative breast cancers based on Immunogenomic profiling. *Journal of Experimental & Clinical Cancer Research*.

[23] Yoshihara K., Shahmoradgoli M., Martínez E. (2013). Inferring tumour purity and stromal and immune cell admixture from expression data. *Nature Communications*.

[24] Yu G., Wang L. G., Han Y., He Q. Y. (2012). clusterProfiler: an R package for comparing biological themes among gene Clusters. *Omics: a journal of integrative biology*.

[25] Newman A. M., Liu C. L., Green M. R. (2015). Robust enumeration of cell subsets from tissue expression profiles. *Nature Methods*.

[26] Langfelder P., Horvath S. (2008). WGCNA: an R package for weighted correlation network analysis. *BMC Bioinformatics*.

[27] Chin C. H., Chen S. H., Wu H. H., Ho C. W., Ko M. T., Lin C. Y. (2014). cytoHubba: identifying hub objects and sub-networks from complex interactome. *BMC Systems Biology*.

[28] Moore K. J., Sheedy F. J., Fisher E. A. (2013). Macrophages in atherosclerosis: a dynamic balance. *Nature Reviews Immunology*.

[29] Ginhoux F., Schultze J. L., Murray P. J., Ochando J., Biswas S. K. (2016). New insights into the multidimensional concept of macrophage ontogeny, activation and function. *Nature Immunology*.

[30] Frostegård J., Ulfgren A. K., Nyberg P. (1999). Cytokine expression in advanced human atherosclerotic plaques: dominance of pro-inflammatory (Th1) and macrophage-stimulating cytokines. *Atherosclerosis*.

[31] Huang S. C., Everts B., Ivanova Y. (2014). Cell-intrinsic lysosomal lipolysis is essential for alternative activation of macrophages. *Nature Immunology*.

[32] Pennock N. D., White J. T., Cross E. W., Cheney E. E., Tamburini B. A., Kedl R. M. (2013). T cell responses: naive to memory and everything in between. *Advances in Physiology Education*.

[33] Tabas I., Lichtman A. H. (2017). Monocyte-macrophages and T cells in atherosclerosis. *Immunity*.

[34] Kyaw T., Winship A., Tay C. (2013). Cytotoxic and proinflammatory CD8+ T lymphocytes promote development of vulnerable atherosclerotic plaques in apoE-deficient mice. *Circulation*.

[35] Kolbus D., Ramos O. H., Berg K. E. (2010). CD8+ T cell activation predominate early immune responses to hypercholesterolemia in Apoe^−^(/)^−^ mice. *BMC Immunology*.

[36] van Duijn J., Kuiper J., Slütter B. (2018). The many faces of CD8+ T cells in atherosclerosis. *Current Opinion in Lipidology*.

[37] Tse K., Tse H., Sidney J., Sette A., Ley K. (2013). T cells in atherosclerosis. *International Immunology*.

[38] Liu Z. D., Wang L., Lu F. H. (2012). Increased Th17 cell frequency concomitant with decreased Foxp3+ Treg cell frequency in the peripheral circulation of patients with carotid artery plaques. *Inflammation Research*.

[39] Ait-Oufella H., Salomon B. L., Potteaux S. (2006). Natural regulatory T cells control the development of atherosclerosis in mice. *Nature Medicine*.

[40] Jonasson L., Holm J., Skalli O., Bondjers G., Hansson G. K. (1986). Regional accumulations of T cells, macrophages, and smooth muscle cells in the human atherosclerotic plaque. *Arteriosclerosis*.

[41] Bot I., Shi G. P., Kovanen P. T. (2015). Mast cells as effectors in atherosclerosis. *Arteriosclerosis, Thrombosis, and Vascular Biology*.

[42] Kritas S. K., Caraffa A., Antinolfi P. (2014). IgE generation and mast cell activation. *European Journal of Inflammation*.

[43] Xiaogang W., Dingbiao Z., Weidong Y., Huaiyin S., Xiaoming Z., Xiaochen Z. (2002). Distribution of mast cells in carotid atherosclerotic plaque. *PLA medical journal*.

[44] Lappalainen H., Laine P., Pentikäinen M. O., Sajantila A., Kovanen P. T. (2004). Mast cells in neovascularized human coronary plaques store and secrete basic fibroblast growth factor, a potent angiogenic mediator. *Arteriosclerosis, Thrombosis, and Vascular Biology*.

[45] den Dekker W. K., Tempel D., Bot I. (2012). Mast cells induce vascular smooth muscle cell apoptosis via a toll-like receptor 4 activation pathway. *Arteriosclerosis, Thrombosis, and Vascular Biology*.

[46] Selathurai A., Deswaerte V., Kanellakis P. (2014). Natural killer (NK) cells augment atherosclerosis by cytotoxic-dependent mechanisms. *Cardiovascular Research*.

[47] Nour-Eldine W., Joffre J., Zibara K. (2018). Genetic depletion or Hyperresponsiveness of natural killer cells do not affect atherosclerosis development. *Circulation Research*.

[48] Miller J. P., Allman D. (2003). The decline in B lymphopoiesis in aged mice reflects loss of very early B-lineage precursors. *Journal of Immunology*.

[49] Frasca D., Landin A. M., Lechner S. C. (2008). Aging down-regulates the transcription factor E2A, activation-induced cytidine deaminase, and Ig class switch in human B cells. *Journal of Immunology*.

[50] Caligiuri G., Nicoletti A., Poirier B., Hansson G. K. (2002). Protective immunity against atherosclerosis carried by B cells of hypercholesterolemic mice. *The Journal of Clinical Investigation*.

[51] Major A. S., Fazio S., Linton M. F. (2002). B-lymphocyte deficiency increases atherosclerosis in LDL receptor-null mice. *Arteriosclerosis, Thrombosis, and Vascular Biology*.

[52] Xia N., Hasselwander S., Reifenberg G. (2021). B lymphocyte-deficiency in mice causes vascular dysfunction by inducing neutrophilia. *Biomedicine*.

[53] Combadière C., Potteaux S., Rodero M. (2008). Combined inhibition of CCL2, CX3CR1, and CCR5 abrogates Ly6C(hi) and Ly6C(lo) monocytosis and almost abolishes atherosclerosis in hypercholesterolemic mice. *Circulation*.

[54] Winter C., Silvestre-Roig C., Ortega-Gomez A. (2018). Chrono-pharmacological targeting of the CCL2-CCR2 Axis ameliorates atherosclerosis. *Cell Metabolism*.

[55] Chang T. T., Yang H. Y., Chen C., Chen J. W. (2020). CCL4 inhibition in atherosclerosis: effects on plaque stability, endothelial cell adhesiveness, and macrophages activation. *International Journal of Molecular Sciences*.

[56] Cao F., Castrillo A., Tontonoz P., Re F., Byrne G. I. (2007). Chlamydia pneumoniae--induced macrophage foam cell formation is mediated by Toll-like receptor 2. *Infection and Immunity*.

[57] Chukkapalli S. S., Velsko I. M., Rivera-Kweh M. F., Larjava H., Lucas A. R., Kesavalu L. (2017). Global TLR2 and 4 deficiency in mice impacts bone resorption, inflammatory markers and atherosclerosis to polymicrobial infection. *Molecular Oral Microbiology*.

[58] Triantafilou M., Gamper F. G., Lepper P. M. (2007). Lipopolysaccharides from atherosclerosis-associated bacteria antagonize TLR4, induce formation of TLR2/1/CD36 complexes in lipid rafts and trigger TLR2-induced inflammatory responses in human vascular endothelial cells. *Cellular Microbiology*.

[59] Wang X. X., Lv X. X., Wang J. P. (2013). Blocking TLR2 activity diminishes and stabilizes advanced atherosclerotic lesions in apolipoprotein E-deficient mice. *Acta Pharmacologica Sinica*.

[60] Lee G. L., Yeh C. C., Wu J. Y. (2019). TLR2 promotes vascular smooth muscle cell Chondrogenic differentiation and consequent calcification via the concerted actions of Osteoprotegerin suppression and IL-6-mediated RANKL induction. *Arteriosclerosis, Thrombosis, and Vascular Biology*.

[61] Everett B. M., MacFadyen J. G., Thuren T., Libby P., Glynn R. J., Ridker P. M. (2020). Inhibition of interleukin-1*β* and reduction in Atherothrombotic cardiovascular events in the CANTOS trial. *Journal of the American College of Cardiology*.

[62] Xia M., Wu Q., Chen P., Qian C. (2021). Regulatory T cell-related gene biomarkers in the deterioration of atherosclerosis. *Frontiers in Cardiovascular Medicine*.

[63] Su W., Zhao Y., Wei Y., Zhang X., Ji J., Yang S. (2021). Exploring the pathogenesis of psoriasis complicated with atherosclerosis via microarray data analysis. *Frontiers in Immunology*.

[64] Pan Y., Yu C., Huang J., Rong Y., Chen J., Chen M. (2020). Bioinformatics analysis of vascular RNA-seq data revealed hub genes and pathways in a novel Tibetan minipig atherosclerosis model induced by a high fat/cholesterol diet. *Lipids in Health and Disease*.

[65] Clarke R., Du H., Kurmi O. (2017). Burden of carotid artery atherosclerosis in Chinese adults: Implications for future risk of cardiovascular diseases. *European Journal of Preventive Cardiology*.

[66] Fowkes F. G., Rudan D., Rudan I. (2013). Comparison of global estimates of prevalence and risk factors for peripheral artery disease in 2000 and 2010: a systematic review and analysis. *Lancet*.

[67] Chen M., Chen S., Yang D. (2021). Weighted gene co-expression network analysis identifies crucial genes mediating progression of carotid plaque. *Frontiers in Physiology*.

[68] Wang C. H., Shi H. H., Chen L. H., Li X. L., Cao G. L., Hu X. F. (2019). Identification of key lncRNAs associated with atherosclerosis progression based on public datasets. *Frontiers in Genetics*.

[69] Chen S., Yang D., Liu Z. (2020). Crucial gene identification in carotid atherosclerosis based on peripheral blood mononuclear cell (PBMC) data by weighted (gene) correlation network analysis (WGCNA). *Medical Science Monitor*.

[70] Wang L., Gao B., Wu M., Yuan W., Liang P., Huang J. (2021). Profiles of immune cell infiltration in carotid artery atherosclerosis based on gene expression data. *Frontiers in Immunology*.

