# Correction to “Analysis of Immune and Inflammation Characteristics of Atherosclerosis from Different Sample Sources”

**DOI:** 10.1155/omcl/9821314

**Published:** 2025-10-17

H. NIE, C. Yan, W. Zhou, and T. Li, “Analysis of Immune and Inflammation Characteristics of Atherosclerosis from Different Sample Sources,” *Oxidative Medicine and Cellular Longevity* 2022 (2022): 5491038, https://doi.org/10.1155/2022/5491038.

In the article titled “Analysis of Immune and Inflammation Characteristics of Atherosclerosis from Different Sample Sources,” there was an error in Affiliation 1. Department of Stem Cell Biology, Atomic Bomb Diseases Institute, Nagasaki University, China, which was incorrect. The corrected affiliation appears below:

Department of Stem Cell Biology, Atomic Bomb Diseases Institute, Nagasaki University, Japan

We apologize for this error.

