# Supplementary material for: Analysis of Immune and Inflammation Characteristics of Atherosclerosis from Different Sample Sources
Source: Oxid Med Cell Longev. 2022 Apr 25;2022:5491038. doi: 10.1155/2022/5491038 (PMC9060985; doi:10.1155/2022/5491038)
Supplement: Supplementary Materials — Supplementary Figure1: Analysis flow chart of this work. Supplementary Figure 2. A: The fusion and de-batch effect of five carotid artery plaque data sets B: The fusion and de-batch effect of two lower extremity atherosclerotic artery data sets. Supplementary Figure 3 A: Heatmap of GSE28829 (including 16 advanced and 13 early carotid plaques) obtained using single-sample gene set enrichment analysis (ssGSEA) B: Heatmap of GSE43292 (including 32 carotid plaques and 32 control samples) obtained using ssGSEA C: Heatmap of GSE100927 (including 29 carotid atherosclerotic artery samples and 12 control samples) obtained using ssGSEA D: Principal component analysis (PCA) of GSE28829 (according to ssGSEA score) E: PCA analysis of GSE43292 (according to ssGSEA score) F: PCA analysis of GSE100927 (according to ssGSEA score). Supplementary Figure 4 A: The volcano map of the differences in gene analysis between the high- and low-immune groups in carotid plaque samples B: The volcano map of the differences in gene analysis between the high- and low-immune groups in peripheral plaque samples C: The volcano map of the differences in gene analysis between the high- and low-immune groups in carotid atherosclerotic artery samples D: The volcano map of the differences in gene analysis between the high- and low-immune groups in lower extremity atherosclerotic artery samples. Supplementary Figure 5 A: Proportion of 22 types of immune cell infiltration in GSE28829 (including 16 advanced and 13 early carotid plaques) B: Differential expression of 22 immune cells in GSE28829 (including 16 advanced and 13 early carotid plaques) between the high and low immune groups C: Selection process of the soft threshold using weighted gene co-expression network analysis (WGCNA) in the carotid plaque group D: Selection process of the soft threshold using WGCNA in the peripheral plaque group E: Selection process of the soft threshold using WGCNA in the carotid atherosclerotic artery group F: Selection pro [file 5491038.f1.zip › Supplementary Table 1.docx]

| Supplementary Table 1: three hundred and eight genes in the blue module |
| --- |

| ABCA5 |
| --- |
| ABCA7 |
| ABCB6 |
| ADAM20 |
| ADAMTSL1 |
| ADAMTSL5 |
| ADCK1 |
| ADCY3 |
| ADH1A |
| ADRA1A |
| ADRA1B |
| AGPAT1 |
| AGPAT3 |
| AKNA |
| ALKBH8 |
| ANGEL1 |
| ANKRD16 |
| APOL1 |
| ARHGAP11A |
| ARHGAP18 |
| ARHGAP19 |
| ASPHD2 |
| ATG16L2 |
| ATG4C |
| ATP10A |
| ATP13A2 |
| ATP1B3 |
| ATP2B1 |
| ATP6V1A |
| B4GALNT1 |
| B4GALNT3 |
| BAX |
| BCAP31 |
| BCL2L11 |
| BDKRB1 |
| C14orf37 |
| C1S |
| C3orf52 |
| C3orf62 |
| C9orf72 |
| CACNB4 |
| CALCR |
| CASP4 |
| CASQ1 |
| CASZ1 |
| CATSPER1 |
| CCDC18 |
| CCDC80 |
| CCR3 |
| CD40 |
| CD44 |
| CD7 |
| CDC14A |
| CDK5R1 |
| CEL |
| CFB |
| CHAC1 |
| CHST7 |
| CLN8 |
| CMTM5 |
| CORO6 |
| CPEB1 |
| CPNE5 |
| CPT1A |
| CRLF3 |
| CRYBB2 |
| CSPG5 |
| CTRL |
| CXorf57 |
| CYP2C8 |
| CYP7B1 |
| DCLRE1B |
| DDX58 |
| DERL1 |
| DGKQ |
| DHCR7 |
| DHRS3 |
| DNMT1 |
| DOK1 |
| DPP10 |
| DPP3 |
| DSC3 |
| DUSP2 |
| ELAVL4 |
| EME2 |
| EMILIN1 |
| ENTPD1 |
| ENTPD7 |
| EPHB6 |
| F8 |
| FAM111B |
| FAM60A |
| FBLN2 |
| FBXL13 |
| FEN1 |
| FES |
| FGD1 |
| FMNL2 |
| FOXP2 |
| FSIP2 |
| FUT1 |
| FUT8 |
| GAB3 |
| GCK |
| GGA2 |
| GGH |
| GINS2 |
| GKN1 |
| GLI1 |
| GNA14 |
| GNAL |
| GNRH1 |
| GPR135 |
| GRB2 |
| GRK4 |
| GRK6 |
| GRM6 |
| GYG2 |
| HAS3 |
| HERC6 |
| HRC |
| HS2ST1 |
| HSPBAP1 |
| IFIT1 |
| IFIT2 |
| IFNGR1 |
| IL17B |
| IMPG1 |
| INPP4A |
| IQCG |
| ITGB3 |
| ITGB3BP |
| ITM2C |
| IVNS1ABP |
| KCNE4 |
| KCNMA1 |
| KIAA1958 |
| KIF23 |
| KLHDC8B |
| KLHL10 |
| KRT19 |
| LACE1 |
| LCN1 |
| LEFTY1 |
| LGALS3 |
| LIN9 |
| LITAF |
| LLGL1 |
| LRFN3 |
| LRRC39 |
| LRRC6 |
| LRRK1 |
| LYPD3 |
| MAD2L1 |
| MAGEE2 |
| MAP2K6 |
| MARS2 |
| MCM8 |
| MDGA1 |
| MED25 |
| MEFV |
| MFSD7 |
| MICALL1 |
| MIER2 |
| MLKL |
| MLLT11 |
| MMP28 |
| MOV10L1 |
| MXD1 |
| MYH3 |
| MYO3A |
| NAGPA |
| NBL1 |
| NDE1 |
| NDUFC1 |
| NFATC2 |
| NLRC5 |
| NLRP9 |
| NMNAT2 |
| NOG |
| NOS1AP |
| NPAS3 |
| NPPC |
| NRP1 |
| NRP2 |
| NT5DC2 |
| NUDT13 |
| OVGP1 |
| OXR1 |
| PAPSS2 |
| PARP12 |
| PARP14 |
| PCDH15 |
| PDCD1LG2 |
| PDK1 |
| PDSS1 |
| PDZD4 |
| PEBP4 |
| PGK1 |
| PIM3 |
| PPP1R16A |
| PPP2R2C |
| PPP3R1 |
| PRDM11 |
| PRICKLE1 |
| PRIM1 |
| PROSC |
| PSD3 |
| PSORS1C1 |
| PSTPIP2 |
| PTK7 |
| RAB15 |
| RAB3IP |
| RAPGEFL1 |
| RASSF7 |
| REEP2 |
| RGS2 |
| RIPK2 |
| RNF122 |
| RNF130 |
| RNF135 |
| RNF168 |
| RNF24 |
| ROBO3 |
| ROPN1B |
| RORB |
| RREB1 |
| RRP12 |
| SAMD9 |
| SAMD9L |
| SCAMP2 |
| SCN1B |
| SEC14L1 |
| SERPINB9 |
| SGK2 |
| SH3BP1 |
| SHKBP1 |
| SIPA1L2 |
| SLC22A2 |
| SLC25A21 |
| SLC25A35 |
| SLC4A3 |
| SLC8A3 |
| SLFN13 |
| SMAD6 |
| SORD |
| SPHK2 |
| SPTB |
| SPTLC2 |
| SRGAP1 |
| SRGAP2 |
| ST3GAL6 |
| STAC2 |
| STEAP3 |
| STMN1 |
| STOX2 |
| STRBP |
| STRN4 |
| STXBP2 |
| STYXL1 |
| SYT17 |
| TBC1D7 |
| TBC1D9 |
| TBC1D9B |
| TBX1 |
| TEKT3 |
| TEX12 |
| TFPT |
| TIMP1 |
| TLCD1 |
| TLE3 |
| TLX1 |
| TMEM61 |
| TMEM97 |
| TMTC4 |
| TNXB |
| TPMT |
| TSPAN1 |
| TTLL11 |
| TUBD1 |
| TWF2 |
| TYR |
| UBQLNL |
| WDR73 |
| WDR90 |
| WFDC10B |
| WNT2B |
| WNT5B |
| ZAR1 |
| ZBTB46 |
| ZDHHC23 |
| ZFP14 |
| ZFP30 |
| ZFYVE26 |
| ZFYVE28 |
| ZMIZ1 |
| ZNF367 |
| ZNF470 |
| ZNF483 |
| ZNF502 |
| ZNF530 |
| ZNF568 |
| ZNF620 |
| ZNF667 |
| ZNF675 |
| ZNF713 |
| ZNF786 |
| ZNRF2 |
