# Supplementary material for: Analysis of Immune and Inflammation Characteristics of Atherosclerosis from Different Sample Sources
Source: Oxid Med Cell Longev. 2022 Apr 25;2022:5491038. doi: 10.1155/2022/5491038 (PMC9060985; doi:10.1155/2022/5491038)
Supplement: Supplementary Materials — Supplementary Figure1: Analysis flow chart of this work. Supplementary Figure 2. A: The fusion and de-batch effect of five carotid artery plaque data sets B: The fusion and de-batch effect of two lower extremity atherosclerotic artery data sets. Supplementary Figure 3 A: Heatmap of GSE28829 (including 16 advanced and 13 early carotid plaques) obtained using single-sample gene set enrichment analysis (ssGSEA) B: Heatmap of GSE43292 (including 32 carotid plaques and 32 control samples) obtained using ssGSEA C: Heatmap of GSE100927 (including 29 carotid atherosclerotic artery samples and 12 control samples) obtained using ssGSEA D: Principal component analysis (PCA) of GSE28829 (according to ssGSEA score) E: PCA analysis of GSE43292 (according to ssGSEA score) F: PCA analysis of GSE100927 (according to ssGSEA score). Supplementary Figure 4 A: The volcano map of the differences in gene analysis between the high- and low-immune groups in carotid plaque samples B: The volcano map of the differences in gene analysis between the high- and low-immune groups in peripheral plaque samples C: The volcano map of the differences in gene analysis between the high- and low-immune groups in carotid atherosclerotic artery samples D: The volcano map of the differences in gene analysis between the high- and low-immune groups in lower extremity atherosclerotic artery samples. Supplementary Figure 5 A: Proportion of 22 types of immune cell infiltration in GSE28829 (including 16 advanced and 13 early carotid plaques) B: Differential expression of 22 immune cells in GSE28829 (including 16 advanced and 13 early carotid plaques) between the high and low immune groups C: Selection process of the soft threshold using weighted gene co-expression network analysis (WGCNA) in the carotid plaque group D: Selection process of the soft threshold using WGCNA in the peripheral plaque group E: Selection process of the soft threshold using WGCNA in the carotid atherosclerotic artery group F: Selection pro [file 5491038.f1.zip › Supplementary Table 2.docx]

| Supplementary Table 2: two thousand one hundred and twenty genes in the turquoise module |
| --- |

| AADAC |
| --- |
| AADAT |
| AASS |
| ABCA1 |
| ABCB1 |
| ABCB4 |
| ABCC3 |
| ABCC4 |
| ABCG1 |
| ABHD12 |
| ABHD6 |
| ABI3 |
| ABI3BP |
| ABP1 |
| ABRA |
| ACACB |
| ACADSB |
| ACAP1 |
| ACBD7 |
| ACCN2 |
| ACOT4 |
| ACOT8 |
| ACP2 |
| ACP6 |
| ACPP |
| ACSBG1 |
| ACSBG2 |
| ACSL5 |
| ACSL6 |
| ACSM5 |
| ACTA1 |
| ADA |
| ADAM12 |
| ADAM17 |
| ADAM20 |
| ADAM28 |
| ADAM29 |
| ADAM32 |
| ADAM8 |
| ADAMTS1 |
| ADAMTS17 |
| ADAMTS4 |
| ADAMTS6 |
| ADAMTS9 |
| ADAP2 |
| ADCY5 |
| ADIPOQ |
| ADM |
| ADORA2B |
| ADORA3 |
| ADPRH |
| ADRA2B |
| ADRA2C |
| ADRB1 |
| ADRB2 |
| ADRBK2 |
| ADSSL1 |
| AFF1 |
| AGBL3 |
| AGPAT9 |
| AHSA2 |
| AIF1 |
| AIF1L |
| AIM1 |
| AK5 |
| AKR1E2 |
| ALDH1A1 |
| ALDH1A3 |
| ALDH7A1 |
| ALDH8A1 |
| ALOX15B |
| ALOX5 |
| ALOX5AP |
| ALPK2 |
| ALS2CL |
| ALS2CR11 |
| ALS2CR12 |
| ALS2CR8 |
| AMDHD1 |
| AMICA1 |
| AMIGO1 |
| AMOT |
| AMPD3 |
| AMPH |
| AMT |
| ANGPT1 |
| ANGPT2 |
| ANGPTL1 |
| ANGPTL4 |
| ANK1 |
| ANKRD22 |
| ANKRD29 |
| ANKRD58 |
| ANKRD7 |
| ANKS1B |
| ANLN |
| ANPEP |
| AOAH |
| AP1B1 |
| AP1G2 |
| AP1S1 |
| AP1S3 |
| APBA2 |
| APBB1IP |
| APCDD1 |
| APOB |
| APOBEC3B |
| APOBEC3C |
| APOBEC3D |
| APOBEC3G |
| APOL1 |
| APOLD1 |
| AQP3 |
| ARAP2 |
| ARHGAP15 |
| ARHGAP18 |
| ARHGAP22 |
| ARHGAP25 |
| ARHGAP30 |
| ARHGAP9 |
| ARHGEF4 |
| ARID5A |
| ARL11 |
| ARL15 |
| ARL4C |
| ARL6 |
| ARMC4 |
| ARPC1B |
| ARRB1 |
| ARRB2 |
| ARRDC3 |
| ARRDC4 |
| ART3 |
| AS3MT |
| ASAH1 |
| ASB2 |
| ASCL2 |
| ASF1B |
| ASGR1 |
| ASGR2 |
| ASMT |
| ASPM |
| ASPRV1 |
| ASRGL1 |
| ASTN2 |
| ATF3 |
| ATN1 |
| ATOH8 |
| ATP1B1 |
| ATP1B2 |
| ATP1B3 |
| ATP2B2 |
| ATP6V0B |
| ATP6V1B2 |
| ATP6V1F |
| ATP8B2 |
| ATP8B3 |
| ATP8B4 |
| AURKA |
| AURKAPS1 |
| AURKB |
| AXIN2 |
| B3GALTL |
| B3GNT5 |
| B3GNT7 |
| B4GALNT3 |
| BAALC |
| BACH2 |
| BATF |
| BATF2 |
| BAX |
| BBOX1 |
| BCAM |
| BCAT1 |
| BCL11A |
| BCL11B |
| BCL3 |
| BCL7A |
| BCO2 |
| BDH2 |
| BHMT2 |
| BIN2 |
| BIRC3 |
| BIVM |
| BLID |
| BLM |
| BLNK |
| BMF |
| BMP2 |
| BMP7 |
| BMP8B |
| BRCA2 |
| BRSK2 |
| BST1 |
| BST2 |
| BTBD11 |
| BTBD16 |
| BTC |
| BTG2 |
| BTK |
| BTN3A2 |
| BUB1 |
| BUB1B |
| BZRAP1 |
| BZW1 |
| C10orf125 |
| C10orf58 |
| C10orf79 |
| C10orf81 |
| C11orf45 |
| C11orf46 |
| C11orf82 |
| C12orf48 |
| C12orf49 |
| C12orf5 |
| C13orf15 |
| C13orf18 |
| C13orf31 |
| C13orf34 |
| C14orf104 |
| C14orf128 |
| C14orf39 |
| C14orf82 |
| C15orf21 |
| C15orf33 |
| C15orf42 |
| C16orf54 |
| C16orf75 |
| C17orf44 |
| C17orf48 |
| C17orf57 |
| C17orf60 |
| C17orf87 |
| C18orf54 |
| C18orf56 |
| C19orf18 |
| C19orf59 |
| C1GALT1 |
| C1QA |
| C1QB |
| C1QC |
| C1QTNF2 |
| C1QTNF3 |
| C1QTNF8 |
| C1orf103 |
| C1orf106 |
| C1orf118 |
| C1orf162 |
| C1orf163 |
| C1orf182 |
| C1orf226 |
| C1orf229 |
| C1orf38 |
| C20orf103 |
| C20orf197 |
| C20orf200 |
| C20orf26 |
| C21orf34 |
| C22orf34 |
| C2orf65 |
| C3AR1 |
| C3orf62 |
| C4BPB |
| C4orf39 |
| C5 |
| C5AR1 |
| C5orf13 |
| C5orf20 |
| C5orf35 |
| C5orf54 |
| C5orf56 |
| C6orf105 |
| C6orf115 |
| C6orf155 |
| C6orf173 |
| C6orf192 |
| C6orf195 |
| C6orf97 |
| C7orf29 |
| C7orf53 |
| C7orf57 |
| C7orf63 |
| C8orf46 |
| C8orf77 |
| C9orf100 |
| C9orf130 |
| C9orf167 |
| C9orf38 |
| C9orf47 |
| C9orf66 |
| C9orf71 |
| C9orf72 |
| C9orf93 |
| CAB39L |
| CACNA2D3 |
| CACNA2D4 |
| CACNB1 |
| CADM1 |
| CADM3 |
| CALR |
| CAMK4 |
| CAMP |
| CAPG |
| CAPN11 |
| CARD16 |
| CARD6 |
| CARD9 |
| CASC5 |
| CASP1 |
| CASP5 |
| CASQ1 |
| CASZ1 |
| CATSPER1 |
| CATSPER2 |
| CAV3 |
| CCDC109B |
| CCDC11 |
| CCDC121 |
| CCDC122 |
| CCDC14 |
| CCDC141 |
| CCDC89 |
| CCL17 |
| CCL2 |
| CCL20 |
| CCL22 |
| CCL23 |
| CCL26 |
| CCL28 |
| CCL5 |
| CCL7 |
| CCNA1 |
| CCNA2 |
| CCNB1 |
| CCNB2 |
| CCNE1 |
| CCNE2 |
| CCNT1 |
| CCR1 |
| CCR2 |
| CCR5 |
| CCR6 |
| CCR7 |
| CCRL1 |
| CD109 |
| CD14 |
| CD163 |
| CD177 |
| CD180 |
| CD1A |
| CD1B |
| CD1D |
| CD2 |
| CD200 |
| CD22 |
| CD226 |
| CD244 |
| CD247 |
| CD274 |
| CD300A |
| CD300C |
| CD300E |
| CD300LB |
| CD300LF |
| CD33 |
| CD37 |
| CD38 |
| CD3E |
| CD3G |
| CD4 |
| CD40 |
| CD40LG |
| CD48 |
| CD5 |
| CD5L |
| CD6 |
| CD68 |
| CD69 |
| CD72 |
| CD79A |
| CD80 |
| CD83 |
| CD84 |
| CD86 |
| CD8A |
| CD8B |
| CD96 |
| CDC20 |
| CDC45L |
| CDC6 |
| CDCA2 |
| CDCA3 |
| CDCA5 |
| CDCA8 |
| CDCP1 |
| CDH1 |
| CDH23 |
| CDH3 |
| CDH6 |
| CDH8 |
| CDKL2 |
| CDKN2C |
| CDKN3 |
| CDNF |
| CDON |
| CDS1 |
| CEBPA |
| CEBPD |
| CECR1 |
| CECR6 |
| CELSR1 |
| CENPA |
| CENPE |
| CENPF |
| CENPH |
| CENPK |
| CENPN |
| CENPQ |
| CENPV |
| CEP152 |
| CEP70 |
| CEP72 |
| CETP |
| CFD |
| CGNL1 |
| CH25H |
| CHD5 |
| CHD7 |
| CHEK1 |
| CHI3L2 |
| CHKA |
| CHN2 |
| CHST13 |
| CHST2 |
| CIITA |
| CISH |
| CIT |
| CITED2 |
| CKAP2 |
| CKAP2L |
| CKS2 |
| CLDN1 |
| CLDN23 |
| CLEC12A |
| CLEC2B |
| CLEC4A |
| CLEC4C |
| CLEC4D |
| CLEC5A |
| CLEC7A |
| CLECL1 |
| CLIP4 |
| CLLU1OS |
| CLN6 |
| CLN8 |
| CLYBL |
| CMAH |
| CMPK2 |
| CMTM7 |
| CNIH3 |
| CNKSR1 |
| CNR2 |
| CNTN3 |
| CNTN6 |
| COL10A1 |
| COL22A1 |
| COL27A1 |
| COLEC12 |
| COMMD6 |
| COQ2 |
| CORO1A |
| CORO2A |
| CORO2B |
| CORO6 |
| CORO7 |
| COTL1 |
| CPAMD8 |
| CPEB1 |
| CPM |
| CPS1 |
| CPT1A |
| CPVL |
| CR1 |
| CRTAC1 |
| CRTAM |
| CRYBB1 |
| CSAD |
| CSDC2 |
| CSF1R |
| CSF2RA |
| CSF2RB |
| CSF3 |
| CSN1S1 |
| CSRP2 |
| CST7 |
| CSTB |
| CTH |
| CTLA4 |
| CTNNAL1 |
| CTSB |
| CTSC |
| CTSD |
| CTSK |
| CTSL1 |
| CTSL2 |
| CTSS |
| CTSW |
| CTSZ |
| CTTNBP2 |
| CUX2 |
| CX3CL1 |
| CXADR |
| CXCL13 |
| CXCL16 |
| CXCL6 |
| CXCR3 |
| CXorf15 |
| CXorf21 |
| CXorf65 |
| CYB5R4 |
| CYBA |
| CYBB |
| CYGB |
| CYP1B1 |
| CYP27A1 |
| CYP27B1 |
| CYP2B7P1 |
| CYP2E1 |
| CYP2S1 |
| CYP39A1 |
| CYP3A5 |
| DAB2 |
| DAPK1 |
| DAPP1 |
| DAZL |
| DCT |
| DEF6 |
| DENND1C |
| DENND2D |
| DEPDC1B |
| DEPDC6 |
| DEPDC7 |
| DERL1 |
| DFNB31 |
| DGCR8 |
| DGKE |
| DGKG |
| DHCR7 |
| DHDH |
| DHFRL1 |
| DHRS9 |
| DIRAS2 |
| DISC1 |
| DLEU2L |
| DLEU7 |
| DLG2 |
| DLG3 |
| DLGAP5 |
| DMP1 |
| DMXL2 |
| DNA2 |
| DNAH7 |
| DNAJB1 |
| DNAJB14 |
| DNAJB4 |
| DNAJB5 |
| DNAJC6 |
| DNASE2B |
| DOCK2 |
| DOCK4 |
| DOCK8 |
| DOK2 |
| DOK3 |
| DOK6 |
| DOPEY1 |
| DPEP2 |
| DPP4 |
| DPPA4 |
| DRAM1 |
| DSC2 |
| DSC3 |
| DSCC1 |
| DTL |
| DTX1 |
| DTX3L |
| DUSP1 |
| DUSP10 |
| DUSP19 |
| DUSP2 |
| DUSP5 |
| DUSP5P |
| DUSP8 |
| DYNC1I1 |
| DYNC2H1 |
| DYSF |
| DYX1C1 |
| E2F2 |
| E2F7 |
| E2F8 |
| EAF2 |
| EBI3 |
| ECHDC1 |
| ECM1 |
| ECT2 |
| EDEM2 |
| EFHA2 |
| EFHD2 |
| EFS |
| EGFL6 |
| EGFR |
| EGR2 |
| EGR3 |
| EIF2C2 |
| ELAVL4 |
| ELF5 |
| ELMO1 |
| ELOVL4 |
| EMILIN2 |
| EMR1 |
| EMR2 |
| ENC1 |
| ENG |
| ENOX1 |
| ENPP2 |
| ENPP5 |
| ENTPD3 |
| EOMES |
| EPB41L3 |
| EPB41L5 |
| EPB49 |
| EPHA1 |
| EPHA2 |
| EPHB2 |
| EPHX1 |
| EPSTI1 |
| ERAP2 |
| ERBB2 |
| EREG |
| ERMN |
| ERP27 |
| ESCO2 |
| ETNK2 |
| EVI2A |
| EVI2B |
| EXO1 |
| EXPH5 |
| EYA2 |
| EZH2 |
| F10 |
| F11R |
| F2RL1 |
| FAIM3 |
| FAM101A |
| FAM102B |
| FAM105A |
| FAM107B |
| FAM110A |
| FAM110C |
| FAM134B |
| FAM149A |
| FAM151B |
| FAM154B |
| FAM163A |
| FAM181B |
| FAM19A1 |
| FAM20A |
| FAM20B |
| FAM26D |
| FAM3B |
| FAM40B |
| FAM49A |
| FAM54A |
| FAM71C |
| FAM78A |
| FAM83H |
| FAM89A |
| FAM90A1 |
| FAM96A |
| FANCG |
| FANCI |
| FAR2 |
| FASLG |
| FAT4 |
| FBLN7 |
| FBP1 |
| FBXO27 |
| FBXO30 |
| FBXO32 |
| FBXO40 |
| FBXO41 |
| FBXO6 |
| FCN3 |
| FCRL5 |
| FCRLA |
| FCRLB |
| FDX1 |
| FERMT3 |
| FGD3 |
| FGD5 |
| FGD6 |
| FGF11 |
| FGF13 |
| FGF18 |
| FGF9 |
| FGFBP3 |
| FGR |
| FIGN |
| FILIP1 |
| FJX1 |
| FKBP11 |
| FKBP1B |
| FKBP5 |
| FKBP7 |
| FKBP8 |
| FLNC |
| FLRT1 |
| FLRT3 |
| FLT3 |
| FMNL1 |
| FNDC5 |
| FOLR2 |
| FOS |
| FOXD2 |
| FOXF1 |
| FOXG1 |
| FOXM1 |
| FOXO4 |
| FPR1 |
| FRMD4B |
| FRMD7 |
| FSIP1 |
| FSIP2 |
| FTH1 |
| FUCA1 |
| FUT8 |
| FXYD5 |
| FYB |
| FZD3 |
| G0S2 |
| GAA |
| GABRA4 |
| GABRB1 |
| GABRB2 |
| GAD1 |
| GADD45B |
| GAL |
| GALNT12 |
| GALNT14 |
| GALNT6 |
| GAP43 |
| GAPT |
| GAS2 |
| GAS2L3 |
| GAS7 |
| GATA3 |
| GBGT1 |
| GBP5 |
| GCHFR |
| GCK |
| GCNT1 |
| GCNT2 |
| GCOM1 |
| GDA |
| GDF9 |
| GEN1 |
| GFI1 |
| GFPT2 |
| GGH |
| GHR |
| GIMAP2 |
| GIMAP4 |
| GINS2 |
| GINS3 |
| GINS4 |
| GJA4 |
| GJB2 |
| GK |
| GKAP1 |
| GLA |
| GLB1L |
| GLI2 |
| GLUL |
| GMFG |
| GMIP |
| GMPR |
| GNA15 |
| GNG2 |
| GNGT2 |
| GNLY |
| GNRH1 |
| GOLGA7B |
| GOLM1 |
| GPC2 |
| GPER |
| GPLD1 |
| GPM6B |
| GPR1 |
| GPR116 |
| GPR125 |
| GPR137B |
| GPR155 |
| GPR160 |
| GPR171 |
| GPR18 |
| GPR19 |
| GPR34 |
| GPR37 |
| GPR63 |
| GPR64 |
| GPR65 |
| GPR68 |
| GPR82 |
| GPR84 |
| GPR85 |
| GPRASP1 |
| GPRC5A |
| GPRIN3 |
| GPSM3 |
| GPX1 |
| GRAMD4 |
| GREM2 |
| GRHL1 |
| GRIA1 |
| GRIA3 |
| GRIK5 |
| GRIN2D |
| GRIN3A |
| GRIP2 |
| GRK4 |
| GRM1 |
| GRM6 |
| GRN |
| GSC |
| GSDMA |
| GSDMC |
| GSTM3 |
| GSTO2 |
| GTF2IRD2 |
| GTSE1 |
| GTSF1 |
| GUCA1B |
| GUCY1A3 |
| GUCY1B3 |
| GULP1 |
| GVIN1 |
| GZMA |
| GZMH |
| GZMK |
| HAMP |
| HAVCR2 |
| HBEGF |
| HCG2P7 |
| HCK |
| HCLS1 |
| HCP5 |
| HCST |
| HDX |
| HEATR1 |
| HEATR3 |
| HELLS |
| HERC6 |
| HES2 |
| HESX1 |
| HEXA |
| HFE |
| HHAT |
| HHEX |
| HIF3A |
| HIRA |
| HIST1H3C |
| HIST2H2AC |
| HJURP |
| HK2 |
| HK3 |
| HLA-DMB |
| HLA-DOA |
| HLA-DOB |
| HLA-DPB1 |
| HLA-DPB2 |
| HLA-DRB1 |
| HMGA1 |
| HMGB3 |
| HMHA1 |
| HMMR |
| HN1 |
| HOMER2 |
| HOPX |
| HOXA10 |
| HOXA13 |
| HOXA2 |
| HOXA3 |
| HOXA4 |
| HOXA5 |
| HOXA9 |
| HOXB2 |
| HOXB3 |
| HOXB6 |
| HOXC11 |
| HOXC5 |
| HOXC6 |
| HOXC8 |
| HPD |
| HPGDS |
| HPSE |
| HRC |
| HS3ST1 |
| HS3ST3A1 |
| HS3ST3B1 |
| HSD11B1 |
| HSD17B3 |
| HSD3B7 |
| HSPA12A |
| HSPA4 |
| HSPA4L |
| HSPA6 |
| HSPB2 |
| HSPB8 |
| HTR2A |
| HTR4 |
| HTR7 |
| HTRA4 |
| HYMAI |
| ICA1 |
| ICAM1 |
| ICOS |
| IER3 |
| IER5L |
| IFI27 |
| IFI30 |
| IFI44 |
| IFI44L |
| IFIT1 |
| IFIT3 |
| IFT81 |
| IGF2BP3 |
| IGFBP1 |
| IGFBP6 |
| IGSF21 |
| IGSF6 |
| IGSF8 |
| IKBKE |
| IKZF1 |
| IKZF3 |
| IL10 |
| IL10RA |
| IL11RA |
| IL16 |
| IL17RA |
| IL17RD |
| IL18 |
| IL18BP |
| IL18R1 |
| IL1A |
| IL1RAP |
| IL21R |
| IL24 |
| IL27RA |
| IL28RA |
| IL2RB |
| IL2RG |
| IL32 |
| IL4I1 |
| IL7 |
| INADL |
| INPP5B |
| INPP5D |
| INSM1 |
| INTU |
| IQGAP2 |
| IRAK3 |
| IRF1 |
| IRF4 |
| IRF5 |
| IRF7 |
| IRF8 |
| IRX2 |
| IRX5 |
| ISG20 |
| ITGA10 |
| ITGAD |
| ITGAL |
| ITGAM |
| ITGAX |
| ITGB2 |
| ITGB7 |
| ITGB8 |
| ITIH4 |
| ITK |
| JAK3 |
| JAKMIP1 |
| JPH2 |
| JUB |
| JUN |
| JUNB |
| KAZALD1 |
| KBTBD12 |
| KBTBD8 |
| KCNA3 |
| KCNB1 |
| KCNB2 |
| KCND3 |
| KCNE1 |
| KCNE2 |
| KCNE3 |
| KCNJ10 |
| KCNJ11 |
| KCNJ2 |
| KCNJ5 |
| KCNK13 |
| KCNK3 |
| KCNN2 |
| KCNN4 |
| KCNQ1 |
| KCNQ4 |
| KCTD16 |
| KDM1B |
| KIAA0125 |
| KIAA0319 |
| KIAA0408 |
| KIAA0754 |
| KIAA1107 |
| KIAA1161 |
| KIAA1211 |
| KIAA1217 |
| KIAA1244 |
| KIAA1377 |
| KIAA1486 |
| KIAA1598 |
| KIAA1683 |
| KIAA1712 |
| KIAA1755 |
| KIAA1841 |
| KIAA1958 |
| KIF11 |
| KIF15 |
| KIF18B |
| KIF20A |
| KIF21B |
| KIFC1 |
| KL |
| KLF1 |
| KLF2 |
| KLF4 |
| KLF5 |
| KLHDC10 |
| KLHL10 |
| KLHL13 |
| KLHL23 |
| KLHL6 |
| KLK1 |
| KLK5 |
| KLKB1 |
| KLRA1 |
| KLRB1 |
| KLRC3 |
| KLRF1 |
| KLRG1 |
| KLRK1 |
| KMO |
| KRR1 |
| KYNU |
| L3MBTL |
| L3MBTL4 |
| LACE1 |
| LACTB |
| LAG3 |
| LAIR1 |
| LAMA3 |
| LAMB3 |
| LAP3 |
| LAPTM5 |
| LAT2 |
| LAX1 |
| LCA5 |
| LCA5L |
| LCK |
| LCP2 |
| LDHAL6A |
| LDLR |
| LDOC1 |
| LECT2 |
| LEF1 |
| LEFTY1 |
| LEP |
| LGALS2 |
| LGI2 |
| LGI4 |
| LGMN |
| LGR4 |
| LGR6 |
| LHCGR |
| LHX2 |
| LHX9 |
| LIF |
| LILRA1 |
| LILRA2 |
| LILRA3 |
| LILRA4 |
| LILRA5 |
| LILRB1 |
| LILRB2 |
| LILRB3 |
| LILRB4 |
| LILRB5 |
| LIMD2 |
| LIMK1 |
| LIMS2 |
| LIPA |
| LIPC |
| LIPG |
| LMNB1 |
| LNP1 |
| LOH3CR2A |
| LONRF3 |
| LPAR1 |
| LPAR4 |
| LPAR5 |
| LPL |
| LRCH2 |
| LRIG3 |
| LRMP |
| LRP2BP |
| LRP5 |
| LRRC25 |
| LRRC3 |
| LRRC33 |
| LRRC36 |
| LRRC39 |
| LRRC48 |
| LRRC4C |
| LRRC6 |
| LRRC66 |
| LRRC8B |
| LRRC8C |
| LRRC8D |
| LRRTM2 |
| LSP1 |
| LST1 |
| LTB |
| LTB4R |
| LTBP4 |
| LXN |
| LY6E |
| LY75 |
| LY86 |
| LY96 |
| LYN |
| LYNX1 |
| LYPD6 |
| MAB21L2 |
| MAD2L1 |
| MAFF |
| MAGEE1 |
| MAGI2 |
| MAGOH |
| MAL |
| MAL2 |
| MALL |
| MAMDC2 |
| MAN2B1 |
| MANEA |
| MAP1LC3C |
| MAP3K8 |
| MAP4K1 |
| MAPK13 |
| MAPKBP1 |
| MARK1 |
| MARVELD3 |
| MAT1A |
| MATK |
| MBNL3 |
| MBOAT2 |
| MCART1 |
| MCART6 |
| MCF2L |
| MCHR1 |
| MCOLN1 |
| MCOLN2 |
| MCOLN3 |
| MCTP1 |
| ME2 |
| MEI1 |
| MEIS1 |
| MEIS2 |
| MELK |
| MEOX2 |
| MEST |
| MFNG |
| MFSD1 |
| MGAT4A |
| MGRN1 |
| MGST1 |
| MID1 |
| MID1IP1 |
| MIPOL1 |
| MKI67 |
| MLF1 |
| MLPH |
| MMAA |
| MME |
| MMP19 |
| MMP2 |
| MMP28 |
| MMP3 |
| MMP8 |
| MN1 |
| MNDA |
| MNS1 |
| MOXD1 |
| MPDU1 |
| MPEG1 |
| MPL |
| MPP1 |
| MPP2 |
| MPZL2 |
| MPZL3 |
| MR1 |
| MRO |
| MRPL39 |
| MS4A1 |
| MS4A14 |
| MS4A4A |
| MS4A6A |
| MS4A6E |
| MS4A7 |
| MSH4 |
| MT1F |
| MTSS1 |
| MTTP |
| MTUS2 |
| MUC20 |
| MX1 |
| MX2 |
| MYC |
| MYCL1 |
| MYD88 |
| MYEF2 |
| MYO16 |
| MYO1F |
| MYO1G |
| MYO3B |
| MYO7A |
| MYOM2 |
| MYOT |
| N6AMT1 |
| NAALADL2 |
| NACC1 |
| NAGS |
| NAIP |
| NANOG |
| NAP1L5 |
| NAPSB |
| NBEAL1 |
| NCAM1 |
| NCAPG |
| NCAPH |
| NCCRP1 |
| NCF2 |
| NCKAP1L |
| NCKAP5 |
| NDC80 |
| NDP |
| NDUFB3 |
| NEB |
| NECAB1 |
| NEDD4L |
| NEFH |
| NEIL3 |
| NEK2 |
| NEK6 |
| NELL2 |
| NET1 |
| NETO2 |
| NFAM1 |
| NFATC4 |
| NFE2L3 |
| NFIL3 |
| NFIX |
| NFKBIE |
| NFKBIZ |
| NGDN |
| NID1 |
| NINJ2 |
| NIPAL4 |
| NIPSNAP3B |
| NKAPL |
| NKG7 |
| NKX3-1 |
| NLGN3 |
| NLRC4 |
| NLRP12 |
| NLRP2 |
| NLRP3 |
| NME5 |
| NOD2 |
| NOTCH2NL |
| NPC1 |
| NPL |
| NPR1 |
| NPTXR |
| NR1H3 |
| NR2E3 |
| NR4A1 |
| NR4A3 |
| NRCAM |
| NRG1 |
| NRIP3 |
| NRP2 |
| NTM |
| NTRK2 |
| NUAK2 |
| NUF2 |
| NUP210 |
| NUP210L |
| NUSAP1 |
| NXPH3 |
| OAS1 |
| OAS2 |
| OAS3 |
| OASL |
| OBFC2A |
| OCA2 |
| OLFM2 |
| OPA3 |
| ORAI1 |
| ORC6L |
| OSBPL11 |
| OSBPL3 |
| OSBPL6 |
| OSCAR |
| OSM |
| OSR2 |
| OSTF1 |
| OVGP1 |
| OXCT2 |
| P2RX4 |
| P2RX5 |
| P2RX7 |
| P2RY10 |
| P2RY12 |
| P2RY13 |
| P2RY8 |
| PACRG |
| PACSIN3 |
| PADI2 |
| PAG1 |
| PAK1 |
| PAK3 |
| PALM |
| PALM2 |
| PANK1 |
| PAPLN |
| PAQR4 |
| PAQR5 |
| PAQR6 |
| PARD3 |
| PARD6B |
| PARP15 |
| PART1 |
| PARVB |
| PARVG |
| PAX3 |
| PAX8 |
| PBK |
| PCDH18 |
| PCDHB11 |
| PCDHB17 |
| PCDHB18 |
| PCDHB3 |
| PCDHB5 |
| PCDHB6 |
| PCDHB7 |
| PCDHB9 |
| PCK2 |
| PCSK5 |
| PCSK6 |
| PCYT1B |
| PDCD1LG2 |
| PDE1C |
| PDE3A |
| PDE3B |
| PDE4B |
| PDE4C |
| PDE4D |
| PDE6B |
| PDE7A |
| PDE8B |
| PDGFB |
| PDP2 |
| PDZD2 |
| PDZD4 |
| PEBP4 |
| PEG3 |
| PELO |
| PER1 |
| PER2 |
| PEX6 |
| PFDN6 |
| PFKFB4 |
| PFN4 |
| PGAP1 |
| PGBD5 |
| PGD |
| PGGT1B |
| PHLDA1 |
| PHOSPHO2 |
| PHYHIP |
| PIGR |
| PIGW |
| PIK3AP1 |
| PIK3CG |
| PIK3R5 |
| PIM1 |
| PIM2 |
| PIPOX |
| PITPNC1 |
| PIWIL2 |
| PKD1L2 |
| PKD2L1 |
| PKIG |
| PLA1A |
| PLA2G15 |
| PLA2G2D |
| PLA2G4A |
| PLAC8L1 |
| PLAG1 |
| PLAU |
| PLAUR |
| PLCB2 |
| PLCB4 |
| PLCD4 |
| PLCG2 |
| PLCH1 |
| PLCL1 |
| PLD3 |
| PLEK |
| PLEK2 |
| PLEKHB2 |
| PLEKHG3 |
| PLEKHH2 |
| PLEKHO2 |
| PLIN2 |
| PLK4 |
| PLP1 |
| PLS1 |
| PLSCR1 |
| PLTP |
| PLXDC1 |
| PLXNA2 |
| PLXNB1 |
| PLXNB3 |
| PLXNC1 |
| PMAIP1 |
| PMFBP1 |
| PNMT |
| PNPLA3 |
| PODN |
| POLQ |
| PPAPDC1A |
| PPARG |
| PPIF |
| PPIL4 |
| PPIL6 |
| PPM1E |
| PPM1F |
| PPM1K |
| PPP1R15A |
| PPP1R3B |
| PRAM1 |
| PRDM1 |
| PRDM5 |
| PREX1 |
| PRF1 |
| PRG2 |
| PRH1 |
| PRICKLE1 |
| PRKAA2 |
| PRKCQ |
| PRKX |
| PRO0628 |
| PROCR |
| PROX1 |
| PRPF39 |
| PRPH |
| PRPH2 |
| PRR11 |
| PRR16 |
| PRRT1 |
| PRRT2 |
| PRSS16 |
| PRTG |
| PRUNE2 |
| PSAT1 |
| PSD3 |
| PSD4 |
| PSMA8 |
| PSMB10 |
| PSMB9 |
| PSTPIP1 |
| PTAFR |
| PTCH1 |
| PTER |
| PTGER2 |
| PTGS1 |
| PTGS2 |
| PTH1R |
| PTN |
| PTPN13 |
| PTPN22 |
| PTPN6 |
| PTPN7 |
| PTPRC |
| PTPRCAP |
| PTPRE |
| PTPRF |
| PTPRJ |
| PTPRO |
| PTTG1 |
| PTX3 |
| PVALB |
| PVRIG |
| PYCARD |
| PYGM |
| PYHIN1 |
| QPCT |
| QPRT |
| RAB11B |
| RAB11FIP2 |
| RAB20 |
| RAB33A |
| RAB36 |
| RAB38 |
| RAB39B |
| RAB3C |
| RAB5C |
| RAB6B |
| RAB7B |
| RAB9B |
| RAC2 |
| RAD51 |
| RAD51AP1 |
| RAD9B |
| RAET1E |
| RAI2 |
| RALGDS |
| RALGPS2 |
| RANBP3L |
| RARRES3 |
| RASD1 |
| RASGEF1A |
| RASGEF1B |
| RASGRP1 |
| RASGRP2 |
| RASGRP3 |
| RASGRP4 |
| RASL10A |
| RASL10B |
| RASSF2 |
| RASSF4 |
| RASSF5 |
| RASSF7 |
| RAVER2 |
| RBKS |
| RBM43 |
| RBM44 |
| RBP4 |
| RCAN3 |
| RDH12 |
| RFPL1S |
| RFX4 |
| RG9MTD2 |
| RG9MTD3 |
| RGPD1 |
| RGS16 |
| RHBDF1 |
| RHBDF2 |
| RHD |
| RHOF |
| RHOH |
| RICTOR |
| RIMS1 |
| RIMS3 |
| RINL |
| RNASE1 |
| RNASE3 |
| RNASE6 |
| RND1 |
| RND2 |
| RNF112 |
| RNF125 |
| RNF128 |
| RNF144B |
| RNF150 |
| RNF152 |
| RNF175 |
| RNF180 |
| RNF215 |
| ROCK1 |
| ROCK2 |
| ROR2 |
| RP2 |
| RPA4 |
| RPGRIP1 |
| RPL37 |
| RPPH1 |
| RPS6KA1 |
| RRAD |
| RSAD2 |
| RSPH9 |
| RTN2 |
| RTP4 |
| RUNDC3B |
| RUNX3 |
| RYR1 |
| S100A9 |
| S1PR3 |
| SALL2 |
| SAMD3 |
| SAMD9 |
| SAMD9L |
| SAMSN1 |
| SASH3 |
| SBDSP |
| SBK1 |
| SCAMP5 |
| SCARB1 |
| SCIN |
| SCO2 |
| SDC1 |
| SDC3 |
| SDCBP |
| SDHAP3 |
| SDS |
| SEC14L5 |
| SELP |
| SELPLG |
| SEMA3B |
| SEMA4A |
| SEMA4D |
| SEMA4G |
| SEMA6B |
| SERINC2 |
| SERP2 |
| SERPINA1 |
| SERPINI1 |
| SERTAD1 |
| SERTAD3 |
| SERTAD4 |
| SFMBT2 |
| SFTPD |
| SGCA |
| SGCE |
| SGCG |
| SGK2 |
| SGOL2 |
| SGSM1 |
| SH2D1A |
| SH2D1B |
| SH3BGR |
| SH3BP2 |
| SH3GL2 |
| SH3GL3 |
| SHCBP1 |
| SIDT1 |
| SIGLEC1 |
| SIGLEC10 |
| SIGLEC11 |
| SIGLEC12 |
| SIGLEC16 |
| SIGLEC5 |
| SIGLEC7 |
| SIGLEC8 |
| SIGLEC9 |
| SIGLECP3 |
| SIK1 |
| SILV |
| SIM2 |
| SIRPA |
| SIRPB1 |
| SIRPG |
| SIT1 |
| SIX1 |
| SIX2 |
| SIX5 |
| SKA1 |
| SKAP1 |
| SLA |
| SLA2 |
| SLAMF1 |
| SLAMF6 |
| SLAMF7 |
| SLAMF9 |
| SLC10A4 |
| SLC10A7 |
| SLC12A3 |
| SLC12A7 |
| SLC12A8 |
| SLC15A2 |
| SLC15A3 |
| SLC16A6 |
| SLC19A1 |
| SLC1A3 |
| SLC22A1 |
| SLC22A15 |
| SLC22A16 |
| SLC22A18 |
| SLC22A18AS |
| SLC22A4 |
| SLC23A3 |
| SLC24A4 |
| SLC25A19 |
| SLC25A23 |
| SLC25A27 |
| SLC28A2 |
| SLC28A3 |
| SLC29A3 |
| SLC2A1 |
| SLC2A12 |
| SLC2A3 |
| SLC2A5 |
| SLC31A1 |
| SLC31A2 |
| SLC35F1 |
| SLC36A1 |
| SLC38A4 |
| SLC38A6 |
| SLC39A8 |
| SLC43A3 |
| SLC44A5 |
| SLC45A3 |
| SLC46A2 |
| SLC47A1 |
| SLC4A10 |
| SLC4A11 |
| SLC4A4 |
| SLC4A8 |
| SLC6A12 |
| SLC6A4 |
| SLC6A6 |
| SLC6A8 |
| SLC6A9 |
| SLC7A10 |
| SLC7A11 |
| SLC7A5 |
| SLC7A7 |
| SLC7A8 |
| SLC7A9 |
| SLC9A3R2 |
| SLC9A7 |
| SLCO1C1 |
| SLCO2B1 |
| SLCO4A1 |
| SLCO5A1 |
| SLIT2 |
| SLPI |
| SMARCD1 |
| SMPDL3A |
| SMPX |
| SMTN |
| SNAI1 |
| SNAI3 |
| SNAP25 |
| SNAP91 |
| SNAPC1 |
| SNCA |
| SNCAIP |
| SNORA66 |
| SNRPE |
| SNRPN |
| SNTA1 |
| SNX10 |
| SNX31 |
| SNX5 |
| SOCS3 |
| SOD2 |
| SOHLH2 |
| SOX13 |
| SOX5 |
| SOX6 |
| SP140 |
| SP4 |
| SP6 |
| SPAG4 |
| SPAG5 |
| SPAG8 |
| SPC25 |
| SPDYE1 |
| SPEG |
| SPHK1 |
| SPHKAP |
| SPI1 |
| SPIB |
| SPINT1 |
| SPN |
| SPOCD1 |
| SPOCK2 |
| SPON2 |
| SRD5A3 |
| SRGAP3 |
| SRPK3 |
| SRPX2 |
| SSH2 |
| SSTR1 |
| SSTR2 |
| ST14 |
| ST3GAL5 |
| ST3GAL6 |
| ST6GALNAC2 |
| ST7OT1 |
| ST8SIA4 |
| STAB1 |
| STAC3 |
| STAG3L2 |
| STAMBPL1 |
| STARD4 |
| STARD8 |
| STARD9 |
| STAT4 |
| STC2 |
| STEAP1 |
| STEAP3 |
| STEAP4 |
| STIL |
| STK10 |
| STK17B |
| STK32A |
| STON1 |
| STOX1 |
| STRA6 |
| STX11 |
| STXBP4 |
| SUCNR1 |
| SULT1C2 |
| SUSD1 |
| SUSD2 |
| SUSD3 |
| SUSD4 |
| SV2B |
| SYCP2 |
| SYDE2 |
| SYK |
| SYNE2 |
| SYNGAP1 |
| SYNGR2 |
| SYNGR3 |
| SYPL2 |
| SYT2 |
| SYTL3 |
| TAC1 |
| TACC2 |
| TACC3 |
| TACR1 |
| TAF4 |
| TAGAP |
| TBC1D10C |
| TBC1D2 |
| TBL1X |
| TBX21 |
| TBX3 |
| TBXAS1 |
| TCEA3 |
| TCF7 |
| TCHH |
| TCIRG1 |
| TCL1A |
| TCN2 |
| TCTEX1D1 |
| TDO2 |
| TDRD9 |
| TENC1 |
| TES |
| TF |
| TFAP2C |
| TFCP2L1 |
| TFEC |
| TFRC |
| TGFA |
| THAP9 |
| THSD4 |
| TIAM1 |
| TIAM2 |
| TIFAB |
| TIGD7 |
| TIMD4 |
| TIMP4 |
| TLE1 |
| TLR1 |
| TLR2 |
| TLR3 |
| TLR4 |
| TLR5 |
| TLR6 |
| TLR7 |
| TLR8 |
| TM4SF19 |
| TM6SF1 |
| TMC6 |
| TMC8 |
| TMCC2 |
| TMCC3 |
| TMEFF1 |
| TMEM119 |
| TMEM133 |
| TMEM136 |
| TMEM149 |
| TMEM14B |
| TMEM155 |
| TMEM156 |
| TMEM163 |
| TMEM169 |
| TMEM176A |
| TMEM176B |
| TMEM180 |
| TMEM2 |
| TMEM25 |
| TMEM26 |
| TMEM37 |
| TMEM49 |
| TMEM56 |
| TMEM74 |
| TMEM86A |
| TMEM8B |
| TMPRSS11A |
| TMSB15A |
| TMTC2 |
| TNF |
| TNFAIP2 |
| TNFAIP3 |
| TNFAIP8L2 |
| TNFRSF10A |
| TNFRSF11A |
| TNFRSF17 |
| TNFRSF1B |
| TNFRSF21 |
| TNFRSF8 |
| TNFRSF9 |
| TNFSF13B |
| TNFSF15 |
| TNFSF4 |
| TNFSF8 |
| TNFSF9 |
| TNN |
| TNNC1 |
| TNNC2 |
| TNNI2 |
| TNNT1 |
| TNXB |
| TOM1L1 |
| TOMM7 |
| TOP2A |
| TPPP |
| TPRG1 |
| TPX2 |
| TRAM1L1 |
| TRAT1 |
| TREM2 |
| TREML4 |
| TRIB3 |
| TRIM14 |
| TRIM45 |
| TRIP13 |
| TRNP1 |
| TRO |
| TRPA1 |
| TRPC4 |
| TRPC6 |
| TSC22D1 |
| TSHB |
| TSLP |
| TSPAN13 |
| TSPAN15 |
| TSPAN33 |
| TSPYL2 |
| TTC18 |
| TTC22 |
| TTC25 |
| TTC39A |
| TTC39C |
| TTK |
| TTYH3 |
| TUBB2B |
| TUBB3 |
| TXK |
| TXLNB |
| TXNDC6 |
| TYROBP |
| UAP1L1 |
| UBASH3A |
| UBE2C |
| UBE2T |
| UBQLNL |
| UBXN11 |
| UCP2 |
| UPP1 |
| USP6 |
| UTS2 |
| VAMP8 |
| VANGL2 |
| VASH2 |
| VAV1 |
| VDR |
| VEGFA |
| VENTX |
| VEPH1 |
| VMO1 |
| VNN1 |
| VNN2 |
| VSIG2 |
| VSIG4 |
| VWA3B |
| WAS |
| WASF3 |
| WBSCR17 |
| WDR52 |
| WDR67 |
| WDR70 |
| WISP1 |
| WISP3 |
| WNK3 |
| WNT2 |
| WNT5A |
| WNT5B |
| WSCD1 |
| XAF1 |
| XRCC2 |
| YES1 |
| ZBTB16 |
| ZBTB46 |
| ZC3H12B |
| ZCCHC2 |
| ZCWPW2 |
| ZDHHC11 |
| ZDHHC15 |
| ZFAND1 |
| ZFAT |
| ZFP112 |
| ZFP2 |
| ZFP28 |
| ZFP36 |
| ZFP37 |
| ZFYVE9 |
| ZMAT1 |
| ZMYM1 |
| ZMYND12 |
| ZMYND15 |
| ZNF10 |
| ZNF107 |
| ZNF117 |
| ZNF137 |
| ZNF138 |
| ZNF14 |
| ZNF154 |
| ZNF177 |
| ZNF20 |
| ZNF208 |
| ZNF215 |
| ZNF239 |
| ZNF25 |
| ZNF257 |
| ZNF267 |
| ZNF273 |
| ZNF28 |
| ZNF280C |
| ZNF285A |
| ZNF300 |
| ZNF323 |
| ZNF33B |
| ZNF347 |
| ZNF385A |
| ZNF385D |
| ZNF396 |
| ZNF415 |
| ZNF425 |
| ZNF441 |
| ZNF442 |
| ZNF454 |
| ZNF461 |
| ZNF470 |
| ZNF471 |
| ZNF484 |
| ZNF486 |
| ZNF493 |
| ZNF501 |
| ZNF514 |
| ZNF519 |
| ZNF527 |
| ZNF530 |
| ZNF540 |
| ZNF542 |
| ZNF546 |
| ZNF554 |
| ZNF557 |
| ZNF563 |
| ZNF566 |
| ZNF567 |
| ZNF569 |
| ZNF571 |
| ZNF572 |
| ZNF577 |
| ZNF596 |
| ZNF620 |
| ZNF630 |
| ZNF660 |
| ZNF675 |
| ZNF682 |
| ZNF711 |
| ZNF718 |
| ZNF721 |
| ZNF763 |
| ZNF781 |
| ZNF793 |
| ZNF804A |
| ZNF826 |
| ZNF828 |
| ZNF831 |
| ZNRF3 |
| ZRANB3 |
| ZSCAN12 |
| ZSCAN5A |
| ZWINT |
