# Supplementary material for: Analysis of Immune and Inflammation Characteristics of Atherosclerosis from Different Sample Sources
Source: Oxid Med Cell Longev. 2022 Apr 25;2022:5491038. doi: 10.1155/2022/5491038 (PMC9060985; doi:10.1155/2022/5491038)
Supplement: Supplementary Materials — Supplementary Figure1: Analysis flow chart of this work. Supplementary Figure 2. A: The fusion and de-batch effect of five carotid artery plaque data sets B: The fusion and de-batch effect of two lower extremity atherosclerotic artery data sets. Supplementary Figure 3 A: Heatmap of GSE28829 (including 16 advanced and 13 early carotid plaques) obtained using single-sample gene set enrichment analysis (ssGSEA) B: Heatmap of GSE43292 (including 32 carotid plaques and 32 control samples) obtained using ssGSEA C: Heatmap of GSE100927 (including 29 carotid atherosclerotic artery samples and 12 control samples) obtained using ssGSEA D: Principal component analysis (PCA) of GSE28829 (according to ssGSEA score) E: PCA analysis of GSE43292 (according to ssGSEA score) F: PCA analysis of GSE100927 (according to ssGSEA score). Supplementary Figure 4 A: The volcano map of the differences in gene analysis between the high- and low-immune groups in carotid plaque samples B: The volcano map of the differences in gene analysis between the high- and low-immune groups in peripheral plaque samples C: The volcano map of the differences in gene analysis between the high- and low-immune groups in carotid atherosclerotic artery samples D: The volcano map of the differences in gene analysis between the high- and low-immune groups in lower extremity atherosclerotic artery samples. Supplementary Figure 5 A: Proportion of 22 types of immune cell infiltration in GSE28829 (including 16 advanced and 13 early carotid plaques) B: Differential expression of 22 immune cells in GSE28829 (including 16 advanced and 13 early carotid plaques) between the high and low immune groups C: Selection process of the soft threshold using weighted gene co-expression network analysis (WGCNA) in the carotid plaque group D: Selection process of the soft threshold using WGCNA in the peripheral plaque group E: Selection process of the soft threshold using WGCNA in the carotid atherosclerotic artery group F: Selection pro [file 5491038.f1.zip › Supplementary Table 3.docx]

| Supplementary Table 3: one thousand and forty-two genes in the turquoise module |
| --- |

| ABCA1 |
| --- |
| ABCA7 |
| ABCA8 |
| ABCC3 |
| ABCC9 |
| ABCG1 |
| ACCN4 |
| ACOT4 |
| ACP5 |
| ACRBP |
| ACTN2 |
| ADAM28 |
| ADAM8 |
| ADAMDEC1 |
| ADAMTS14 |
| ADAMTS4 |
| ADAMTS8 |
| ADAMTS9 |
| ADAP2 |
| ADCY7 |
| ADORA2B |
| ADORA3 |
| ADRB2 |
| ADRBK2 |
| AGPAT4-IT1 |
| AGRP |
| AGTR1 |
| AIM2 |
| AK8 |
| AKAP12 |
| AKR1B1 |
| ALDH1L1 |
| ALK |
| ALOX15B |
| ALOX5AP |
| AMICA1 |
| AMPH |
| ANGPTL1 |
| ANGPTL7 |
| ANKRD35 |
| ANKRD58 |
| ANPEP |
| ANXA3 |
| ANXA8L2 |
| AOAH |
| AOC4 |
| AOX1 |
| APBB1IP |
| APCDD1 |
| APOBR |
| APOC1 |
| APOC2 |
| APOD |
| APOE |
| AQP7P3 |
| AQP9 |
| ARHGAP25 |
| ARHGAP30 |
| ARHGAP9 |
| ARL4C |
| ARRB2 |
| ARRDC4 |
| ASB16 |
| ASCL2 |
| ASPA |
| ASPHD1 |
| ASPM |
| ATG16L2 |
| ATP6V0D2 |
| ATP6V1B2 |
| AXIN2 |
| BATF |
| BCAT1 |
| BCHE |
| BCL2A1 |
| BDKRB1 |
| BDKRB2 |
| BEX2 |
| BEX5 |
| BID |
| BIN2 |
| BIRC5 |
| BLNK |
| BTK |
| BVES |
| C11orf35 |
| C11orf75 |
| C13orf15 |
| C13orf33 |
| C14orf132 |
| C14orf34 |
| C15orf27 |
| C15orf48 |
| C16orf54 |
| C16orf89 |
| C1QA |
| C1QB |
| C1QC |
| C1QTNF4 |
| C1orf162 |
| C1orf38 |
| C1orf93 |
| C2 |
| C21orf96 |
| C3AR1 |
| C3orf70 |
| C4orf48 |
| C5AR1 |
| C5orf20 |
| C6orf115 |
| C6orf174 |
| C6orf192 |
| C7orf41 |
| C9orf139 |
| C9orf167 |
| CA12 |
| CA2 |
| CACNA2D4 |
| CACNB2 |
| CAMK2N1 |
| CAPG |
| CARD16 |
| CARD9 |
| CASP1 |
| CASQ1 |
| CASQ2 |
| CATSPER1 |
| CCDC146 |
| CCDC30 |
| CCL15 |
| CCL18 |
| CCL3 |
| CCL3L3 |
| CCL4 |
| CCL5 |
| CCL7 |
| CCNB2 |
| CCR1 |
| CCR2 |
| CCR5 |
| CCR7 |
| CCRL2 |
| CD109 |
| CD14 |
| CD163 |
| CD163L1 |
| CD180 |
| CD248 |
| CD27 |
| CD28 |
| CD300A |
| CD300C |
| CD300LF |
| CD33 |
| CD36 |
| CD37 |
| CD38 |
| CD3D |
| CD3G |
| CD40LG |
| CD5 |
| CD52 |
| CD53 |
| CD6 |
| CD68 |
| CD74 |
| CD82 |
| CD83 |
| CD84 |
| CD96 |
| CDCA8 |
| CDCP1 |
| CDH19 |
| CDH23 |
| CDH3 |
| CDKN2A |
| CDON |
| CDT1 |
| CEBPA |
| CEBPE |
| CECR1 |
| CERS1 |
| CHI3L1 |
| CHIT1 |
| CHODL |
| CHRDL1 |
| CILP |
| CKB |
| CKS2 |
| CLDN23 |
| CLEC11A |
| CLEC4A |
| CLEC5A |
| CLMP |
| CLN6 |
| CMTM3 |
| CMTM7 |
| CNIH3 |
| CNR1 |
| CNTN1 |
| CNTN3 |
| CNTN4 |
| COL18A1 |
| COL4A3 |
| CORO1A |
| CORO2A |
| CORO7 |
| COTL1 |
| CPE |
| CPNE5 |
| CPNE7 |
| CPVL |
| CRISPLD1 |
| CRISPLD2 |
| CRTAC1 |
| CRTAM |
| CSF1R |
| CSF2RA |
| CSF3 |
| CSF3R |
| CSK |
| CSRNP1 |
| CSTB |
| CTSB |
| CTSD |
| CTSH |
| CTSK |
| CTSL1 |
| CTSL1P8 |
| CTSL2 |
| CTSS |
| CTSZ |
| CX3CR1 |
| CXCL10 |
| CXCL14 |
| CXCL16 |
| CXCR2P1 |
| CXCR4 |
| CXCR6 |
| CXCR7 |
| CYBA |
| CYBB |
| CYP27A1 |
| CYP27B1 |
| CYP2S1 |
| CYTH4 |
| CYTIP |
| CYTL1 |
| DAPP1 |
| DARC |
| DBN1 |
| DBP |
| DCDC2C |
| DCN |
| DDX60L |
| DENND1C |
| DENND2D |
| DEPTOR |
| DES |
| DHDH |
| DHRS9 |
| DIRAS1 |
| DNAJB4 |
| DNAJC5B |
| DNASE2B |
| DOCK2 |
| DOCK8 |
| DOK1 |
| DOK3 |
| DPEP2 |
| DPP4 |
| DPRXP4 |
| DPT |
| DRAM1 |
| DRAM2 |
| DSC2 |
| DSP |
| DUSP10 |
| DUSP6 |
| EBI3 |
| ECM1 |
| EFHD2 |
| EGR2 |
| ELL2 |
| ELOVL7 |
| EMILIN2 |
| EMR2 |
| EMX2OS |
| ENTPD2 |
| EVI2A |
| EVI2B |
| FABP5 |
| FAM107A |
| FAM110A |
| FAM180B |
| FAM20A |
| FAM26F |
| FAM49B |
| FAM59A |
| FAM70A |
| FAM71E1 |
| FAM78A |
| FAM96A |
| FAP |
| FASLG |
| FBLN1 |
| FBP1 |
| FBXO41 |
| FCER1A |
| FCER1G |
| FCGBP |
| FCGR1B |
| FCGR2A |
| FCGR2C |
| FCGR3A |
| FCHO1 |
| FCN1 |
| FERMT3 |
| FGD3 |
| FGL2 |
| FGR |
| FILIP1 |
| FKBP15 |
| FLJ46552 |
| FLNC |
| FMNL1 |
| FMO3 |
| FNDC5 |
| FOLR2 |
| FPR1 |
| FST |
| FSTL3 |
| FTL |
| FUCA1 |
| FYB |
| GAA |
| GABRE |
| GAL |
| GALNT5 |
| GALNT6 |
| GALNTL1 |
| GAS1 |
| GAS2L3 |
| GBP5 |
| GCHFR |
| GCNT1 |
| GFRA1 |
| GGTLC1 |
| GIMAP1 |
| GIMAP2 |
| GJC1 |
| GLA |
| GLI1 |
| GLUL |
| GM2A |
| GMFG |
| GMIP |
| GNG11 |
| GNG8 |
| GPC3 |
| GPM6B |
| GPNMB |
| GPR137B |
| GPR160 |
| GPR171 |
| GPR20 |
| GPR34 |
| GPR65 |
| GPR84 |
| GPRC5A |
| GPSM3 |
| GPX1 |
| GRAMD4 |
| GRRP1 |
| GSTM5 |
| GUCA2B |
| GZMA |
| GZMH |
| GZMK |
| HAMP |
| HAND2 |
| HAVCR2 |
| HCAR3 |
| HCK |
| HCLS1 |
| HCST |
| HES2 |
| HES4 |
| HEYL |
| HIST1H1B |
| HIST1H1C |
| HIST2H3A |
| HJURP |
| HK2 |
| HK3 |
| HLA-B |
| HLA-DMA |
| HLA-DMB |
| HLA-DPA1 |
| HLA-DPB1 |
| HLA-DPB2 |
| HLA-DQA1 |
| HLA-DQA2 |
| HLA-DQB1 |
| HLA-DQB2 |
| HLA-DRA |
| HLA-DRB1 |
| HLA-DRB4 |
| HLA-DRB5 |
| HLA-J |
| HMGA1 |
| HMGA1P4 |
| HMHA1 |
| HMMR |
| HMOX1 |
| HN1 |
| HOXB6 |
| HP |
| HPGDS |
| HPR |
| HPSE |
| HPSE2 |
| HRK |
| HS3ST2 |
| HSPA6 |
| HSPB6 |
| HTRA3 |
| HTRA4 |
| HVCN1 |
| IBSP |
| ID1 |
| IFI30 |
| IFI44L |
| IFIT1 |
| IFIT2 |
| IFIT3 |
| IGFBP4 |
| IGFBPL1 |
| IGFLR1 |
| IGJ |
| IGLL1 |
| IGLL5 |
| IGSF6 |
| IL10 |
| IL10RA |
| IL17B |
| IL17D |
| IL17RD |
| IL18 |
| IL1B |
| IL1RN |
| IL21R |
| IL2RB |
| IL2RG |
| IL33 |
| IL4I1 |
| IL6R |
| IL7R |
| INPP5D |
| IQGAP3 |
| IRF5 |
| IRF7 |
| IRF8 |
| ISM1 |
| ITGA4 |
| ITGAD |
| ITGAL |
| ITGAM |
| ITGAX |
| ITGB2 |
| ITGB7 |
| ITM2A |
| ITM2C |
| JAK3 |
| JAKMIP2 |
| JAM2 |
| KANK1 |
| KCNE3 |
| KCNIP3 |
| KCNJ2 |
| KCNJ5 |
| KCNJ8 |
| KCNK17 |
| KCNN4 |
| KIAA0101 |
| KIAA0125 |
| KIAA0226L |
| KIAA1199 |
| KIF5C |
| KLHDC5 |
| KLHDC8B |
| KLHL6 |
| KLK1 |
| KLRB1 |
| KMO |
| KRT14 |
| KRT16P2 |
| KRT18 |
| KRT18P55 |
| KRT19 |
| KRT31 |
| KRT78 |
| LAIR1 |
| LAMB3 |
| LAMP3 |
| LAPTM5 |
| LCK |
| LCP2 |
| LDLR |
| LEF1 |
| LEFTY2 |
| LGALS2 |
| LGALS9 |
| LGALS9C |
| LGI4 |
| LGMN |
| LGR6 |
| LHFPL2 |
| LILRA2 |
| LILRA3 |
| LILRA4 |
| LILRA6 |
| LILRB1 |
| LILRB2 |
| LILRB3 |
| LILRB4 |
| LIMK1 |
| LIMS3L |
| LINC00303 |
| LINC00312 |
| LINC00473 |
| LIPA |
| LIPE |
| LOC100129846 |
| LOC100130811 |
| LOC100131733 |
| LOC100288432 |
| LOC100505504 |
| LOC100505592 |
| LOC100505905 |
| LOC100506115 |
| LOC100506585 |
| LOC100506897 |
| LOC100506948 |
| LOC100506966 |
| LOC100507008 |
| LOC100507165 |
| LOC100507233 |
| LOC100507239 |
| LOC100507334 |
| LOC100507554 |
| LOC100508196 |
| LOC100509553 |
| LOC100652963 |
| LOC100653210 |
| LOC158402 |
| LOC283050 |
| LOC283352 |
| LOC283454 |
| LOC286058 |
| LOC389493 |
| LOC400456 |
| LOC401093 |
| LOC401847 |
| LOC643988 |
| LOC644189 |
| LOC645638 |
| LOC645722 |
| LOC645954 |
| LOC650226 |
| LOC728052 |
| LOC728061 |
| LOC729468 |
| LOC729680 |
| LOXL2 |
| LPAR1 |
| LPAR5 |
| LPL |
| LPXN |
| LRCH2 |
| LRMP |
| LRRC33 |
| LRRC55 |
| LRRN4CL |
| LSP1 |
| LST1 |
| LTB |
| LUM |
| LY6H |
| LY86 |
| LY96 |
| LYN |
| MAF |
| MAFB |
| MAFF |
| MAN2B1 |
| MAP4K1 |
| MAPK13 |
| MAPK4 |
| MAPKAPK3 |
| MARCKS |
| MARCO |
| MATK |
| MCOLN1 |
| MCOLN2 |
| MECOM |
| MEI1 |
| METTL7B |
| MFNG |
| MFSD1 |
| MFSD2A |
| MFSD7 |
| MGST1 |
| MILR1 |
| MIR100HG |
| MLC1 |
| MMD |
| MMP1 |
| MMP12 |
| MMP7 |
| MMP9 |
| MNDA |
| MPEG1 |
| MPP1 |
| MPP6 |
| MREG |
| MS4A14 |
| MS4A4A |
| MS4A6A |
| MS4A7 |
| MSR1 |
| MSX1 |
| MT1E |
| MT1F |
| MT1M |
| MT1X |
| MTUS2 |
| MUSTN1 |
| MX1 |
| MXRA5 |
| MYBPH |
| MYD88 |
| MYO18B |
| MYO1F |
| MYO1G |
| MYO5A |
| MYOT |
| MYOZ2 |
| MZB1 |
| NANOS1 |
| NAP1L2 |
| NAP1L5 |
| NAPSA |
| NBLA00301 |
| NCEH1 |
| NCF1 |
| NCF2 |
| NCF4 |
| NCKAP1L |
| NDUFA4L2 |
| NEURL3 |
| NFAM1 |
| NFATC4 |
| NFIL3 |
| NFIX |
| NFKBIE |
| NGFR |
| NKG7 |
| NLGN1 |
| NLN |
| NLRP12 |
| NLRP3 |
| NNMT |
| NOD2 |
| NOSTRIN |
| NPC1 |
| NPC2 |
| NPL |
| NPTX2 |
| NR1D1 |
| NR4A3 |
| NRIP3 |
| NRK |
| NTRK3 |
| NUAK2 |
| NUSAP1 |
| NXF3 |
| NXPH4 |
| OAS1 |
| OASL |
| OBFC2A |
| OLFML3 |
| OLR1 |
| OSBPL3 |
| OSR1 |
| OSR2 |
| P2RX4 |
| P2RX7 |
| P2RY13 |
| P2RY6 |
| P2RY8 |
| PABPC4L |
| PACSIN1 |
| PAG1 |
| PALM |
| PAQR5 |
| PARP12 |
| PBX4 |
| PCDH11X |
| PCDH11Y |
| PDE6G |
| PDGFB |
| PDPN |
| PDZRN4 |
| PEBP4 |
| PFKFB4 |
| PGD |
| PGM5 |
| PHGDH |
| PHYHIP |
| PI16 |
| PID1 |
| PIK3AP1 |
| PIK3CG |
| PIM2 |
| PITX1 |
| PKD2L1 |
| PKIB |
| PLA2G15 |
| PLA2G2A |
| PLA2G7 |
| PLAU |
| PLBD1 |
| PLCB2 |
| PLCB4 |
| PLCE1 |
| PLD3 |
| PLD5 |
| PLEK2 |
| PLEKHO2 |
| PLIN2 |
| PLIN4 |
| PLP1 |
| PLTP |
| PLXNC1 |
| PLXND1 |
| PMAIP1 |
| PNMT |
| PNOC |
| PNPLA3 |
| PODN |
| PODXL |
| POSTN |
| PPAPDC1A |
| PPARG |
| PPARGC1B |
| PPIF |
| PPL |
| PPP1R9A |
| PRDM1 |
| PRICKLE1 |
| PRRX2 |
| PSAP |
| PTCRA |
| PTGER4 |
| PTGIR |
| PTGS1 |
| PTK2B |
| PTN |
| PTP4A3 |
| PTPN22 |
| PTPN6 |
| PTPN7 |
| PTPRC |
| PTPRCAP |
| PTPRD |
| PTPRO |
| PVRIG |
| PVRL4 |
| PYCARD |
| PYDC1 |
| PYGM |
| RAB42 |
| RAB7B |
| RAC2 |
| RALA |
| RASAL3 |
| RASD1 |
| RASGEF1B |
| RASGRP3 |
| RASL10A |
| RASSF4 |
| RBM47 |
| RBP4 |
| RBP7 |
| RBPMS2 |
| RCAN3 |
| REEP4 |
| RELT |
| RENBP |
| RGMA |
| RGS1 |
| RGS18 |
| RGS19 |
| RHBDF2 |
| RHEBL1 |
| RIMBP3 |
| RIMS4 |
| RNASE1 |
| RNASE6 |
| RNF122 |
| RNF135 |
| RNU105C |
| ROR1 |
| RPS6KA1 |
| RSAD2 |
| RTN1 |
| RTN4R |
| RUNX3 |
| RYR3 |
| S100Z |
| S1PR3 |
| S1PR4 |
| SAMSN1 |
| SAP25 |
| SASH3 |
| SCARA5 |
| SCARB1 |
| SCD |
| SCIN |
| SDIM1 |
| SDS |
| SDSL |
| SEL1L3 |
| SELENBP1 |
| SELL |
| SELM |
| SELPLG |
| SEMA3B |
| SEMA3D |
| SERPINE2 |
| SEZ6L2 |
| SFRP1 |
| SGK1 |
| SGK223 |
| SH3BGR |
| SHISA3 |
| SIGLEC1 |
| SIGLEC15 |
| SIGLEC7 |
| SIGLEC9 |
| SIRPA |
| SKAP1 |
| SKAP2 |
| SLA |
| SLAMF7 |
| SLAMF8 |
| SLAMF9 |
| SLC11A1 |
| SLC15A3 |
| SLC16A10 |
| SLC16A5 |
| SLC16A6 |
| SLC17A9 |
| SLC1A3 |
| SLC22A18 |
| SLC22A18AS |
| SLC22A3 |
| SLC25A19 |
| SLC28A3 |
| SLC29A3 |
| SLC2A9 |
| SLC31A2 |
| SLC36A1 |
| SLC37A2 |
| SLC38A7 |
| SLC43A2 |
| SLC47A1 |
| SLC6A12 |
| SLC7A7 |
| SLCO2B1 |
| SLPI |
| SMAP2 |
| SMPD3 |
| SMPDL3A |
| SMPX |
| SNAI3 |
| SNORA11 |
| SNORA54 |
| SNORD114-3 |
| SNX10 |
| SOCS2 |
| SPI1 |
| SPINK1 |
| SPINT1 |
| SPOCD1 |
| SPON1 |
| SPP1 |
| SRMS |
| SRPK3 |
| SRPX |
| SRRM4 |
| ST14 |
| STAB1 |
| STAC3 |
| STK10 |
| STXBP2 |
| SULT1C2 |
| SUSD1 |
| SYK |
| SYNDIG1 |
| SYNE2 |
| TACC3 |
| TAGAP |
| TBC1D10C |
| TBC1D2 |
| TCAP |
| TCIRG1 |
| TCN2 |
| TFCP2L1 |
| TFEC |
| TGFB3 |
| TGFBI |
| TGFBR3 |
| THBS2 |
| THY1 |
| TIAM1 |
| TIFAB |
| TIMP1 |
| TK1 |
| TLR1 |
| TLR2 |
| TLR7 |
| TM4SF19 |
| TM6SF1 |
| TM7SF4 |
| TMC6 |
| TMEM100 |
| TMEM158 |
| TMEM176A |
| TMEM176B |
| TMEM200A |
| TMEM35 |
| TMEM51 |
| TMEM61 |
| TMEM86A |
| TMEM88 |
| TMSB15A |
| TMSB15B |
| TNC |
| TNF |
| TNFAIP8L2 |
| TNFRSF21 |
| TNNI2 |
| TNNT1 |
| TNNT3 |
| TPST2 |
| TRAF3IP3 |
| TREM1 |
| TREM2 |
| TRGV7 |
| TRIB3 |
| TRIM14 |
| TRIM58 |
| TRPM2 |
| TRPV2 |
| TRPV4 |
| TSKU |
| TSPAN10 |
| TSPAN15 |
| TSPAN2 |
| TSPAN33 |
| TSPAN7 |
| TTLL11 |
| TTYH3 |
| TUBB3 |
| TXNDC3 |
| TYROBP |
| UAP1 |
| UAP1L1 |
| UCP2 |
| UNC13C |
| UNC5B |
| UNC93B1 |
| VAMP8 |
| VAV1 |
| VAV3 |
| VCAM1 |
| VENTX |
| VIPR2 |
| VIT |
| VMO1 |
| VNN1 |
| VNN2 |
| VSIG4 |
| WAS |
| WDR81 |
| WNT11 |
| XLOC_000578 |
| XLOC_000978 |
| XLOC_001215 |
| XLOC_001228 |
| XLOC_001788 |
| XLOC_001855 |
| XLOC_003146 |
| XLOC_003776 |
| XLOC_003784 |
| XLOC_004555 |
| XLOC_004924 |
| XLOC_005062 |
| XLOC_007433 |
| XLOC_008251 |
| XLOC_008644 |
| XLOC_009549 |
| XLOC_009582 |
| XLOC_009932 |
| XLOC_009994 |
| XLOC_010931 |
| XLOC_011331 |
| XLOC_011608 |
| XLOC_011815 |
| XLOC_013436 |
| XLOC_013900 |
| XLOC_013983 |
| XLOC_014161 |
| XLOC_l2_000342 |
| XLOC_l2_002790 |
| XLOC_l2_004840 |
| XLOC_l2_006821 |
| XLOC_l2_007424 |
| XLOC_l2_007731 |
| XLOC_l2_008396 |
| XLOC_l2_009273 |
| XLOC_l2_010897 |
| XLOC_l2_012953 |
| XLOC_l2_013193 |
| XLOC_l2_013301 |
| ZNF300P1 |
| ZNF385A |
| ZNF683 |
| ZNRF3 |
| ZWINT |
