# Supplementary material for: Analysis of Immune and Inflammation Characteristics of Atherosclerosis from Different Sample Sources
Source: Oxid Med Cell Longev. 2022 Apr 25;2022:5491038. doi: 10.1155/2022/5491038 (PMC9060985; doi:10.1155/2022/5491038)
Supplement: Supplementary Materials — Supplementary Figure1: Analysis flow chart of this work. Supplementary Figure 2. A: The fusion and de-batch effect of five carotid artery plaque data sets B: The fusion and de-batch effect of two lower extremity atherosclerotic artery data sets. Supplementary Figure 3 A: Heatmap of GSE28829 (including 16 advanced and 13 early carotid plaques) obtained using single-sample gene set enrichment analysis (ssGSEA) B: Heatmap of GSE43292 (including 32 carotid plaques and 32 control samples) obtained using ssGSEA C: Heatmap of GSE100927 (including 29 carotid atherosclerotic artery samples and 12 control samples) obtained using ssGSEA D: Principal component analysis (PCA) of GSE28829 (according to ssGSEA score) E: PCA analysis of GSE43292 (according to ssGSEA score) F: PCA analysis of GSE100927 (according to ssGSEA score). Supplementary Figure 4 A: The volcano map of the differences in gene analysis between the high- and low-immune groups in carotid plaque samples B: The volcano map of the differences in gene analysis between the high- and low-immune groups in peripheral plaque samples C: The volcano map of the differences in gene analysis between the high- and low-immune groups in carotid atherosclerotic artery samples D: The volcano map of the differences in gene analysis between the high- and low-immune groups in lower extremity atherosclerotic artery samples. Supplementary Figure 5 A: Proportion of 22 types of immune cell infiltration in GSE28829 (including 16 advanced and 13 early carotid plaques) B: Differential expression of 22 immune cells in GSE28829 (including 16 advanced and 13 early carotid plaques) between the high and low immune groups C: Selection process of the soft threshold using weighted gene co-expression network analysis (WGCNA) in the carotid plaque group D: Selection process of the soft threshold using WGCNA in the peripheral plaque group E: Selection process of the soft threshold using WGCNA in the carotid atherosclerotic artery group F: Selection pro [file 5491038.f1.zip › Supplementary Table 4.docx]

| Supplementary Table 4: three hundred and sixty-one genes in the blue module |
| --- |

| AANAT |
| --- |
| ABCA1 |
| ACIN1 |
| ACP5 |
| ADAM8 |
| ADAMDEC1 |
| ADAP2 |
| ADORA3 |
| AGRP |
| AGT |
| AKAP12 |
| ALOX15B |
| ALOX5AP |
| ANKRD58 |
| ANPEP |
| ANXA8L2 |
| APBB1IP |
| APOC1 |
| APOC2 |
| APOE |
| AQP9 |
| ARHGAP9 |
| ARL4C |
| BCAT1 |
| BTK |
| C10orf10 |
| C19orf33 |
| C1QA |
| C1QB |
| C1QC |
| C1QTNF5 |
| C1orf162 |
| C2 |
| C5AR1 |
| CAPG |
| CCDC66 |
| CCL13 |
| CCL18 |
| CCL3 |
| CCL3L3 |
| CCL4 |
| CCL5 |
| CCR1 |
| CCR7 |
| CD14 |
| CD163 |
| CD209 |
| CD300A |
| CD300LF |
| CD33 |
| CD36 |
| CD37 |
| CD3D |
| CD52 |
| CD53 |
| CD68 |
| CD74 |
| CD83 |
| CD84 |
| CD86 |
| CEBPA |
| CEBPE |
| CECR1 |
| CFD |
| CHAD |
| CHGA |
| CISH |
| CLEC4G |
| CLEC4GP1 |
| CLEC5A |
| CLIC3 |
| COL18A1 |
| COL1A1 |
| COL1A2 |
| COL3A1 |
| COL4A2 |
| COL5A2 |
| COLEC11 |
| CORO1A |
| CPNE7 |
| CPVL |
| CSF1R |
| CSTB |
| CTSB |
| CTSD |
| CTSH |
| CTSL1 |
| CTSS |
| CTSZ |
| CXCL10 |
| CYBA |
| CYBB |
| CYP27A1 |
| DENND2D |
| DHRS9 |
| DIRAS1 |
| DMP1 |
| DOCK2 |
| DPEP2 |
| DPEP3 |
| EMILIN2 |
| EMR2 |
| EPSTI1 |
| ESAM |
| F13A1 |
| FAM26F |
| FBP1 |
| FCER1A |
| FCER1G |
| FCGR1B |
| FCGR2A |
| FCGR3A |
| FCGRT |
| FCN1 |
| FERMT2 |
| FERMT3 |
| FGD3 |
| FGR |
| FLNC |
| FLVCR2 |
| FNDC1 |
| FOLR2 |
| FSTL1 |
| FTL |
| FUCA1 |
| FYB |
| FZD4 |
| GAL3ST4 |
| GAPT |
| GBP5 |
| GCHFR |
| GJA4 |
| GM2A |
| GNLY |
| GPNMB |
| GPR137B |
| GPR179 |
| GPR182 |
| GPR65 |
| GZMA |
| GZMB |
| GZMH |
| GZMK |
| HAMP |
| HAND2 |
| HAVCR2 |
| HBA2 |
| HBD |
| HBQ1 |
| HCK |
| HCLS1 |
| HCST |
| HEYL |
| HIST1H1A |
| HK3 |
| HLA-DMA |
| HLA-DMB |
| HLA-DPA1 |
| HLA-DQA1 |
| HLA-DQA2 |
| HLA-DQB1 |
| HLA-DQB2 |
| HLA-DRA |
| HLA-DRB1 |
| HLA-DRB4 |
| HLA-DRB5 |
| HLA-DRB6 |
| HMBOX1 |
| HMOX1 |
| HPGDS |
| HPSE |
| HRK |
| HS3ST1 |
| HS3ST2 |
| HSD17B6 |
| HTRA4 |
| IBSP |
| ID1 |
| IFI30 |
| IGFBP1 |
| IGSF6 |
| IL10 |
| IL10RA |
| IL18 |
| IL2RA |
| IL4I1 |
| IL7R |
| IRF5 |
| IRF8 |
| ITGA5 |
| ITGAM |
| ITGAX |
| ITGB2 |
| ITGB7 |
| KCNK5 |
| KCNN4 |
| KGFLP1 |
| KIR2DL4 |
| KLRB1 |
| KRT79 |
| KRTAP19-2 |
| LAPTM5 |
| LGALS2 |
| LGMN |
| LHFPL2 |
| LILRA2 |
| LILRA4 |
| LILRB1 |
| LILRB3 |
| LILRB4 |
| LIPA |
| LOC348840 |
| LOC400958 |
| LPL |
| LPXN |
| LRRC2 |
| LTB |
| LUM |
| LY86 |
| LYN |
| LYVE1 |
| MAFB |
| MAGEB6 |
| MAPK13 |
| MARCO |
| MATK |
| MDFI |
| ME2 |
| MERTK |
| MFSD1 |
| MMD |
| MMP12 |
| MMP19 |
| MMP7 |
| MMP9 |
| MNDA |
| MPP1 |
| MRC1 |
| MS4A4A |
| MS4A6A |
| MS4A7 |
| MXRA5 |
| MYLK2 |
| MYO1F |
| MYO1G |
| NCF2 |
| NCF4 |
| NCKAP1L |
| NEURL |
| NFAM1 |
| NFIB |
| NINJ2 |
| NKG7 |
| NPL |
| NR1D1 |
| NR2F1 |
| OLR1 |
| OR52E8 |
| OSR2 |
| PAQR5 |
| PCDHGB2 |
| PDE6G |
| PDGFRA |
| PGD |
| PGF |
| PIK3R5 |
| PKD2L1 |
| PLA2G15 |
| PLA2G7 |
| PLAT |
| PLCB2 |
| PLTP |
| PNOC |
| POSTN |
| PROC |
| PSAP |
| PTCRA |
| PTHLH |
| PTP4A3 |
| PTPRO |
| PVRL4 |
| RAB42 |
| RASGEF1B |
| RASL10A |
| RBP1 |
| RBP4 |
| RBP7 |
| REP15 |
| RGS1 |
| RNASE1 |
| RNASE6 |
| RNF150 |
| RPA4 |
| RSPO3 |
| SASH3 |
| SCD |
| SDS |
| SEMA5B |
| SIGLEC1 |
| SIGLEC15 |
| SIGLEC9 |
| SLA |
| SLAMF7 |
| SLAMF8 |
| SLC11A1 |
| SLC15A3 |
| SLC16A10 |
| SLC1A3 |
| SLC31A2 |
| SLC37A2 |
| SLC40A1 |
| SLC7A7 |
| SLCO2B1 |
| SLITRK4 |
| SMARCA4 |
| SNORD38A |
| SNX10 |
| SORL1 |
| SPARCL1 |
| SPEN |
| SPI1 |
| SPP1 |
| ST14 |
| STAB1 |
| STXBP2 |
| SUSD2 |
| SYK |
| TBC1D10C |
| TBC1D2 |
| TBX2 |
| TEF |
| TFRC |
| TGFB3 |
| TGFBI |
| THBS1 |
| TIFAB |
| TIMD4 |
| TLR2 |
| TLR7 |
| TM4SF19 |
| TM7SF4 |
| TMEM176A |
| TMEM176B |
| TMEM51 |
| TNF |
| TNFAIP6 |
| TNFRSF21 |
| TNNT1 |
| TREM1 |
| TREM2 |
| TTYH3 |
| TYROBP |
| UCP2 |
| VAMP8 |
| VAV1 |
| VMO1 |
| VPS18 |
| VSIG4 |
| WFDC2 |
| WWC1 |
| WWC3 |
