# Supplementary material for: Analysis of Immune and Inflammation Characteristics of Atherosclerosis from Different Sample Sources
Source: Oxid Med Cell Longev. 2022 Apr 25;2022:5491038. doi: 10.1155/2022/5491038 (PMC9060985; doi:10.1155/2022/5491038)
Supplement: Supplementary Materials — Supplementary Figure1: Analysis flow chart of this work. Supplementary Figure 2. A: The fusion and de-batch effect of five carotid artery plaque data sets B: The fusion and de-batch effect of two lower extremity atherosclerotic artery data sets. Supplementary Figure 3 A: Heatmap of GSE28829 (including 16 advanced and 13 early carotid plaques) obtained using single-sample gene set enrichment analysis (ssGSEA) B: Heatmap of GSE43292 (including 32 carotid plaques and 32 control samples) obtained using ssGSEA C: Heatmap of GSE100927 (including 29 carotid atherosclerotic artery samples and 12 control samples) obtained using ssGSEA D: Principal component analysis (PCA) of GSE28829 (according to ssGSEA score) E: PCA analysis of GSE43292 (according to ssGSEA score) F: PCA analysis of GSE100927 (according to ssGSEA score). Supplementary Figure 4 A: The volcano map of the differences in gene analysis between the high- and low-immune groups in carotid plaque samples B: The volcano map of the differences in gene analysis between the high- and low-immune groups in peripheral plaque samples C: The volcano map of the differences in gene analysis between the high- and low-immune groups in carotid atherosclerotic artery samples D: The volcano map of the differences in gene analysis between the high- and low-immune groups in lower extremity atherosclerotic artery samples. Supplementary Figure 5 A: Proportion of 22 types of immune cell infiltration in GSE28829 (including 16 advanced and 13 early carotid plaques) B: Differential expression of 22 immune cells in GSE28829 (including 16 advanced and 13 early carotid plaques) between the high and low immune groups C: Selection process of the soft threshold using weighted gene co-expression network analysis (WGCNA) in the carotid plaque group D: Selection process of the soft threshold using WGCNA in the peripheral plaque group E: Selection process of the soft threshold using WGCNA in the carotid atherosclerotic artery group F: Selection pro [file 5491038.f1.zip › Supplementary Table 5.docx]

| **Supplementary Table 5:Correlation analysis between differential immune cells and hub gene in all groups (r value;p value)** | | | | | | | | | | |
| --- | --- | --- | --- | --- | --- | --- | --- | --- | --- | --- |
| **Carotid plaque group** | **Plasma cells** | **T cells CD4 naive** | **T cells follicular helper** | **T cells gamma delta** | **Macrophages M0** | **Dendritic cells resting** | **Mast cells activated** | **T cells regulatory (Tregs)** | **NK cells resting** |  |
| **CD40** | 0.12;0.022 | -0.34;0.001 | 0.17;0.002 | 0.076;0.158 | 0.31;0.001 | 0.15;0.007 | 0.26;0.001 | -0.42;0.001 | -0.33;0.001 |  |
| **NRP1** | 0.17;0.002 | -0.37;0.001 | 0.046;0.4 | 0.012;0.83 | 0.34;0.001 | 0.066;0.221 | 0.22;0.001 | -0.36;0.001 | -0.32;0.001 |  |
| **NRP2** | 0.14;0.011 | -0.35;0.001 | 0.14;0.01 | 0.04;0.465 | 0.4;0.001 | -0.023;0.673 | 0.28;0.001 | -0.39;0.001 | -0.32;0.001 |  |
| **IFNGR1** | 0.15;0.006 | -0.42;0.001 | 0.052;0.335 | 0.093;0.085 | 0.32;0.001 | 0.12;0.027 | 0.25;0.001 | -0.4;0.001 | -0.35;0.001 |  |
| **NFATC2** | 0.2;0.001 | -0.16;0.002 | 0.14;0.007 | 0.2;0.001 | 0.05;0.356 | 0.1;0.061 | 0.038;0.479 | -0.4;0.001 | -0.32;0.001 |  |
|  |  |  |  |  |  |  |  |  |  |  |
| **Peripheral plaque group** | **T cells CD8** | **T cells CD4 memory activated** | **Macrophages M1** | **Mast cells activated** | **Eosinophils** | **B cells naive** | **T cells follicular helper** | **NK cells resting** | **Mast cells resting** |  |
| **PTPRC** | 0.16;0.006 | 0.22;0.001 | 0.13;0.029 | 0.12;0.043 | 0.12;0.04 | -0.41;0.001 | -0.37;0.001 | -0.17;0.005 | -0.27;0.001 |  |
| **CD4** | 0.15;0.011 | 0.13;0.029 | 0.25;0.001 | 0.08;0.172 | 0.032;0.59 | -0.52;0.001 | -0.31;0.001 | -0.22;0.001 | -0.28;0.001 |  |
| **CCL2** | 0.14;0.014 | 0.15;0.011 | 0.24;0.001 | 0.57;0.001 | 0.31;0.001 | -0.29;0.001 | -0.091;0.124 | -0.24;0.001 | -0.51;0.001 |  |
| **TLR2** | 0.085;0.149 | 0.14;0.016 | 0.21;0.001 | 0.14;0.015 | 0.086;0.142 | -0.51;0.001 | -0.29;0.001 | -0.19;0.001 | -0.33；0.001 |  |
| **CD86** | 0.14;0.015 | 0.15;0.009 | 0.25;0.001 | 0.19;0.001 | 0.13;0.022 | -0.53;0.001 | -0.27;0.001 | -0.26;0.001 | -0.35;0.001 |  |
|  |  |  |  |  |  |  |  |  |  |  |
| **Carotid atherosclerotic artery group** | **Macrophages M0** | **Mast cells activated** | **Plasma cells** | **T cells CD4 memory resting** | **Macrophages M1** | **Macrophages M2** | **Mast cells resting** |  |  |  |
| **TNF** | 0.41;0.008 | 0.58;0.001 | -0.54;0.001 | -0.39;0.011 | -0.33;0.035 | -0.28;0.076 | -0.63;0.001 |  |  |  |
| **CCL4** | 0.4;0.009 | 0.9;0.001 | -0.53;0.001 | -0.57;0.001 | -0.58;0.001 | -0.24;0.127 | -0.66;0.001 |  |  |  |
| **PTPRC** | 0.41;0.007 | 0.56;0.001 | -0.39;0.011 | -0.36;0.022 | -0.32;0.039 | -0.51;0.001 | -0.66;0.001 |  |  |  |
| **TLR2** | 0.48;0.001 | 0.74;0.001 | -0.68;0.001 | -0.54;0.001 | -0.67;0.001 | -0.29;0.063 | -0.7;0.001 |  |  |  |
| **IL1B** | 0.52;0.001 | 0.6;0.001 | -0.68;0.001 | -0.61;0.001 | -0.61;0.001 | -0.51;0.001 | -0.67;0.001 |  |  |  |
|  |  |  |  |  |  |  |  |  |  |  |
| **Lower extremity atherosclerotic artery group** | **B cells naive** | **Plasma cells** | **T cells CD8** | **T cells follicular helper** | **T cells gamma delta** | **NK cells resting** | **Macrophages M0** | **Macrophages M1** | **Dendritic cells activated** | **Neutrophils** |
| **HLA-DQA1** | -0.44;0.002 | -0.29;0.047 | 0.42;0.003 | 0.11;0.445 | 0.54;0.001 | -0.3;0.037 | 0.29;0.043 | 0.16;0.281 | -0.21;0.144 | -0.35;0.015 |
| **HLA-DQA2** | 0.11;0.451 | -0.13;0.364 | 0.22;0.134 | -0.22;0.131 | 0.071;0.627 | 0.043;0.768 | 0.17;0.241 | 0.054;0.712 | -0.21;0.154 | -0.089;0.544 |
| **CD3D** | -0.45;0.001 | -0.4;0.004 | -0.075;0.609 | 0.15;0.307 | 0.39;0.006 | -0.013;0.928 | 0.46;0.001 | -0.042;0.772 | -0.17;0.233 | -0.21;0.14 |
| **CD86** | -0.44;0.002 | -0.36;0.012 | -0.086;0.555 | 0.26;0.069 | 0.26;0.071 | 0.009;0.953 | -0.032;0.826 | -0.032;0.826 | -0.084;0.567 | -0.27;0.062 |
| **MMP9** | -0.4;0.004 | -0.51;0.001 | -0.13;0.39 | -0.01;0.946 | 0.33;0.02 | -0.073;0.618 | 0.86;0.001 | -0.42;0.003 | -0.12;0.394 | -0.2;0.168 |
